# Novel Applications of Successive Self-nucleation and Annealing Thermal Fractionation for Polymer Characterization

**DOI:** 10.1021/acsapm.5c04486

**Published:** 2026-02-12

**Authors:** Ricardo A. Pérez-Camargo, Alejandro J. Müller

**Affiliations:** 1 POLYMAT and Department of Polymers and Advanced Materials: Physics, Chemistry and Technology, Faculty of Chemistry, University of the Basque Country UPV/EHU, Donostia-San Sebastián 20018, Spain; 2 IKERBASQUE, Basque Foundation for Science, Plaza Euskadi 5, Bilbao 48009, Spain

**Keywords:** successive self-nucleation and annealing (SSA), thermal fractionation, recycling, polymer topology, sustainability

## Abstract

Successive self-nucleation and annealing (SSA) has evolved into a highly sensitive thermal fractionation protocol capable of resolving subtle lamellar and molecular heterogeneities in semicrystalline polymers. Its relevance has intensified over the past decade as SSA has been applied to sustainable, biobased, biodegradable, and mechanically recycled materials, as well as to systems in which crystallization behavior is tightly linked to circularity, processability, and final performance. In this review, we integrate nearly three decades of SSA developments from a longitudinal perspective, placing particular emphasis on how the role and interpretative power of SSA have progressively expanded in material classes that play a key role in sustainability and recyclability, including aliphatic polyesters and biodegradable copolymers, isodimorphic and mixed-mode random copolymers, nanocomposites with complex interfacial crystallization, and recycled polyolefins and biobased blends. Rather than re-establishing already standard protocols, we briefly revisit the experimental foundations of SSA to provide a self-contained framework that supports the interpretation of these applications, highlighting the influence of key variables such as starting temperatures based on self-nucleation temperature (*T*_*s*_), holding times at *T*_*s*_ (*t*_*s*_), fractionation windows (Δ*T*_*s*_), and scanning rates. We then examine how SSA can elucidate comonomer inclusion/exclusion ratios, topology-driven annealing, interfacial nucleation (supernucleation, prefreezing, and antinucleation), and lamellar reorganization during mechanical recycling. Finally, we highlight SSA as an intermediate crystallization condition that bridges kinetic and thermodynamic regimes, enabling refined lamellar populations and amplifying subtle thermal or polymorphic transitions. By reframing SSA as both an analytical and structure-directing crystallization tool, this review provides an integrated roadmap for researchers aiming to exploit its full potential in the rational design of sustainable, recyclable, and high-performance semicrystalline polymers.

## Graphical abstract

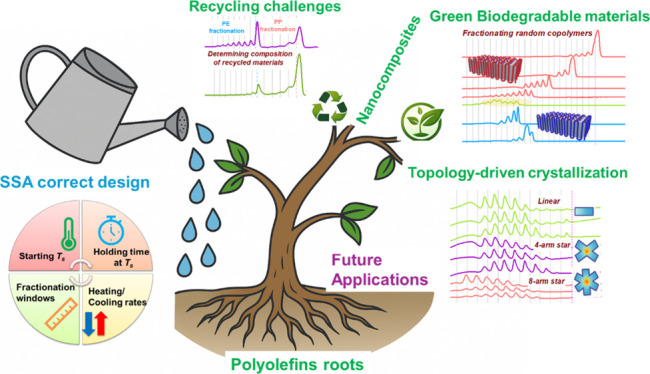

## 1 Introduction

Crystallization-based fractionation techniques are powerful tools to probe the heterogeneity of semicrystalline polymers. Among them, Successive Self-nucleation and Annealing (SSA) stands out for its simplicity (i.e., can be performed in a Differential Scanning Calorimeter, DSC), solvent-free operation, and ability to reveal subtle structural differences without physically separating fractions.^1–4^

While solution-based techniques such as Temperature Rising Elution Fractionation (TREF) and Crystallization Analysis Fractionation (CRYSTAF) remain widely used to determine molar mass distribution and chemically isolate fractions,^5,6^ they are time-consuming, solvent-intensive, and limited to soluble samples.^6^ Thermal fractionation techniques, Step Crystallization (SC)^2–4,7,8^ and SSA overcome these limitations by enabling the analysis of any crystallizable material directly in a DSC, using shorter experimental times and avoiding column plugging or solvent handling.

Since its introduction in 1997 by Müller et al.,^1^ SSA has become a versatile, robust, and scalable method for studying chain heterogeneities in a wide variety of materials. Its power lies in combining isothermal and nonisothermal steps to induce in situ fractionation, which enhances sensitivity to intra- and intermolecular heterogeneities. The ability to use fast scanning rates (up to 50 °C/min in conventional DSC and much higher with Flash Scanning Calorimetry (FSC)) has further expanded its applicability, enabling kinetic studies and dramatically reducing experimental time.^2,3^ With SSA/FSC, the early stages of fractionation can be studied, opening a new research venue.^9,10^

Over nearly three decades, the scope of SSA has progressively expanded beyond its original polyolefin domain. The technique has found renewed relevance in systems where crystallization is more complex or less well understood, such as random copolymers, biodegradable aliphatic polyesters, nanocomposites, and recycled materials, as documented by representative literature compiled in Table 1. This diversification reflects both the maturity of the method and the growing need for fine thermal fractionation tools in sustainable polymer research. As illustrated in Figure 1, the “map” of SSA applications covers the entire evolution of the technique: from its early use in polyolefins to its current role in sustainable, biobased, and multifunctional polymer systems, highlighting its versatility as a bridge between polymer chemistry, morphology, and performance.

**Figure 1:**
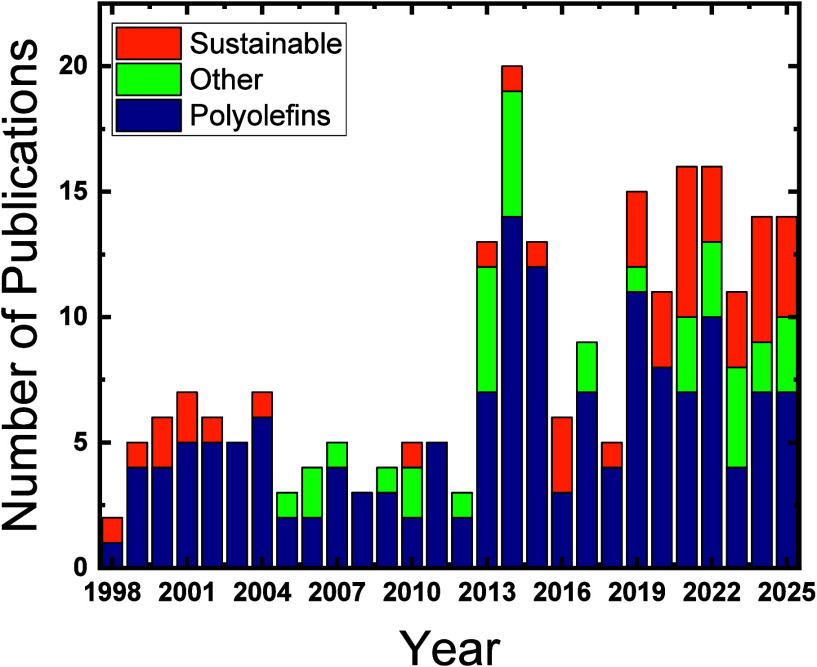
Evolution of the number of publications employing SSA from 1998 to 2025, compiled from the historical SSA literature (including this work (Table 1)) and the major SSA reviews.^2–4^ Publications were classified into three categories: polyolefins, other synthetic polymers, and sustainable/biodegradable systems, following the criteria summarized in Table 1. Early SSA applications were almost exclusively focused on polyolefins. In contrast, in the past decade, SSA has increasingly been adopted to study biodegradable polymers and other nonpolyolefin materials, reflecting the technique’s diversification and expansion beyond its original scope.

**Table 1:** Ten Years (2015–2025) of SSA Applications Devoted to (i) Sustainability: Biodegradable and Recycled Materials, (ii) Polyolefins, and (iii) Other Materials

**authors**	**materials**	**topic**	**year**	**ref**
***sustainability: biodegradable and recycled materials***				
Pérez-Camargo et al.	PCL-*g*-lignin	influence of lignin content on PCL crystallization: supernucleation versus antinucleation effect (hindered annealing during thermal fractionation)	**2015**	^22^
López et al.	*c*-PCL/*l*-PCL blends	threading effects caused by different chain topology on *c*-PCL/*l*-PCL blends	**2016**	^23^
Arandia et al.	PBS-*ran*-PBAz	comonomer exclusion vs inclusion		^24^
Luyt and Gasmi	PLA/PCL blends	crystal size distribution		^25^
Zaldua et al.	*c*- and *l*-PLLA and PDLA	influence of chain topology on lamellar size	**2018**	^26^
Arandia et al.	PBS-*ran*-PBAz	alternative determination of equilibrium melting temperature using SSA maximum melting temperature	**2019**	^27^
Li et al.	POM/PLLA blends	probing spinnability		^28^
Palacios et al.	PEO-*b*-PCL-*b*-PLLA	thermal behavior and crystallization order		^29^
Pérez-Camargo et al.	PBS-*ran*-PBA	comonomer exclusion/inclusion balance under different thermal conditions	**2020**	^15^
Sangroniz et al.	PBS	melt memory effect using SSA+SN experiments		^10^
Carmeli et al.	recycled PE/PP blends	determination of the PP and PE composition in recycled blends		^5^
Fina et al.	PCL/GNP nanopapers	different levels of PCL organization: unoriented and oriented PCL and prefreezing transition	**2021**	^30^
Zhang et al.	PHCU copolymers	co-crystallization behavior: discarding isomorphic or isodimorphic behaviors		^31^
Pérez-Camargo et al.	PCs	even–odd effect		^32^
Pérez-Camargo et al.	PC6 and PC8	solid–solid transitions		^33^
Wang et al.	PLA/PEG/MWCNT	influence of PE and MWCNT ratio on PLA properties		^34^
Yu et al.	PVA-*g*-POSS	change in wafer size measured by SSA		^35^
Sangroniz et al.	poly(ester), poly(ester–ester), and poly(ester–amides)	influence of the intermolecular interactions on SSA profiles	**2022**	^16^
Góra et al.	recycled PP and PE	determination of PP and PE content in recycled materials using fast SSA protocol		^21^
Huang et al.	PVA/talc films	wafer thickness at various melting temperatures		^36^
Fernández-Tena et al.	PCLs	influence of molecular weight on SSA profiles	**2023**	^37^
Zhang et al.	P3HB	influence of stereodefects on long stereoregular crystallizable sequences		^38^
Quinn et al.	P3HB	influence of tacticity defects on crystallizable sequences		^20^
Yang et al.	PLA	intermolecular and intramolecular differences detected by SSA on PLA fractions	**2024**	^39^
Demoor et al.	PCL/graphene nanocomposites	influence of graphene on SSA fractionation (lamellar thickness) of PCL		^40^
Zhao et al.	PCL graphene nanopapers	influence of graphene type and molecular weight on interaction between polymer chains and graphene surface		^41^
Coba-Daza et al.	postconsumer recycled LLDPE/LDPE blends	quantitative method to analyze blends compositions		^42^
Ramírez-Aguilar et al.	PP/discontinued butyl tape	crystalline fractions and nucleating effects		^43^
Gace et al.	P3HBs	influence of stereochemistry (iso- and syndio-rich) and architecture (four-arm star)	**2025**	^44^
Morales et al.	postconsumer PA11/LDPE blends	differences in lamellar structures and influence of processing cycles		^45^
Torres-Rodriguez et al.	PCL*_*x*_* PPDL*_*y*_* copolymers	isodimorphism vs isomorphism identification		^46^
Torres-Rodriguez et al.	polysuccinates, different chain lenghts	even–odd effect		^47^
***polyolefins***				
Kang et al.	PP	lamellar thickness distribution	**2015**	^48^
Xue et al.	branched PE	optimization parameters and comparison with SC		^49^
Xue et al.	LCB-PE	cross-fractionation (SSA and TREF)		^50^
Xue et al.	complex branched LDPE	chain microstructure (SSA and TREF)		^51^
Zheng et al.	iPB	crystallization behavior and sequence length distribution		^52^
Canetti et al.	ethylene/4-methyl-1-pentene copolymres	chain heterogeneity of the copolymer: methylene sequence length, short chain branching, and lamellar thickness		^53^
Xue et al.	PE	methylene sequence length		^54^
Xue et al.	ethylene/1-hexene copolymers	calibration curve: SSA melting vs TREF temperature		^55^
Tong et al.	segmented ethylene-propylene copolymers	chain structure		^56^
Ma et al.	PE	SCB distribution by TREF cross SSA		^57^
Atiqullah et al.	PE	influence of the catalyst on thermal behavior		^58^
Rashedi and Sharif	LLDPE powder from a gas-reactor	comonomer distribution		^59^
Cavallo et al.	LLDPE	SSA using chip-based DSC: influence of *t*_s_ (early stages of fractionation).	**2016**	^9^
Satti et al.	metallocenic ethylene/α-olefin copolymers	studying free-radical post reactions modifications by SSA		^60^
Gumede et al.	LLDPE/Wax blends	plasticization and cocrystallization		^61^
Appiah et al.	PE precision polymers	influence of trans and cis azobenzene defects on the crystallization of PE precision polymers	**2017**	^62^
Ding et al.	Homo and co-PP	study of stereo defects and its distribution		^63^
Zheng et al.	PP copolymers	comonomer content and distribution		^64^
Shandryuk et al.	NB-COE copolymers	crystallization in the multiblock copolymers of norbene and cyclooctene and the appearance of a high-temperature fraction		^65^
Vaezi et al.	BPP	characterization of the soluble part of the reactors blends		^66^
Ogier et al.	EVA	crystalline size distribution and influence of cross-linking		^67^
Ahmadjo et al.	PEs	microstructure of prepared samples		^68^
Li et al.	PE resin	microstructure characterization	**2018**	^69^
Eselem Bungu et al.	branched and linear PE	molecular structure characterization		^70^
Arraez et al.	PP+pro-oxidant	following degradation evolution with SSA		^71^
Li et al.	PE blends	distribution of lamellar thickness and distribution		^72^
Zanchin et al.	ethylene/various α-olefins copolymers	comonomer content and distribution of crystallizable units	**2019**	^73^
Khoshsefat et al.	PE	chain microstructure		^74^
Gholami et al.	PE pipe materials	relationship between creep test failure time and thermal properties		^75^
Eselem Bungu and Pasch	LDPE	structure distribution		^76^
Li et al.	PE	chain structure comparison (TREF vs SSA)		^77^
Létoffé et al.	iPP-*g*-MAH cross-linked with polyether triamine agents	semicrystalline microstructure		^78^
Létoffé et al.	iPP-*g*-MAH cross-linked with polyether triamine agents	impact of the cross-linking		^79^
Leone et al.	ethylene-propylene-1-octene terpolymers	crystallizable sequence length and lamellar thickness		^80^
Hakim et al.	PP	influence of catalyst on the chain microstructure		^81^
Rahmatiyan et al.	ethylene/1,5-hexadiene copolymers	sequence length distribution		^82^
Eselem Bungu et al.	LDPE	branching analysis	**2020**	^83^
Eselem Bungu et al.	PE graft copolymers	molecular structure characterization		^84^
Jiang et al.	Β-iPP	molecular structure characterization		^85^
Tanasi et al.	PE copolymers and nanocomposites	branch distribution		^86^
Liu et al.	HDPE	photodegradation of HDPE under stress		^87^
Groch et al.	E-NB copolymers	influence of catalyst systems on microstructure and thermal properties		^88^
Ghasemi et al.	PP	influence of internal donors on the PP synthesis		^89^
Liu et al.	ethylene homopolymer and ethylene/1-hexene copolymers	influence of catalyst on microstructure study by TREF-SSA techniques		^90^
Li et al.	PE blends	chain microstructure	**2021**	^91^
Zentel et al.	LDPE	microstructure		^92^
Yue et al.	PP + additives	influence of additives in the application of SSA experiments		^93^
Abedini et al.	PE catalyzed in the presence of GNP	number of branches and melting temperature		^94^
Wang et al.	PP	heterogeneity of the crystallizable sequence		^95^
Hu et al.	ethylene copolymers	chain structure		^96^
Denisova et al.	multiblock copolymers of norbonene and Cyclododecene	chain structure		^97^
Wang et al.	mPE	length of crystallizable methylene sequences	**2022**	^98^
Wang et al.	PE pipe resins	molecular chain microstructure		^99^
Urciuoli et al.	ethylene/1-octene multiblock and random copolymers	influence of topological confinement and diluent effect on methylene sequence lengths and distribution		^100^
Zhao and Men	polyolefin elastomer of ethylene/1-octene copolymer (POE) and POE blended with linear PE	methylene sequence length and comonomer distribution		^101^
Li et al.	PE grafted SiO_2_ nanoparticles	confinement and nucleation effect on the grafted PE chains provoked by SiO_2_ NPs		^102^
Hettal et al.	silane-cross-linked low-density PE	effects of radiothermal aging of additive-free silane-cross-linked PE in lamellar thickness distribution		^103^
Jandaghian et al.	monomodal and bimodal ethylene/1-butene copolymers	lamellar thickness distribution depending on the catalyst		^104^
Jandaghian et al.	ethylene and 1-butene copolymers	distribution of SCB of copolymers obtained with various catalyst		^105^
Tenia and Rojas	functionalized PE blended with MWCNTs	lamellar thickness distribution		^106^
Shams et al.	PP	effect of pore diameter on distribution of sequence lenghts and lamellar thickness		^107^
Li et al.	PE/organic-montmorillonite (PE/OMMT) and cross-linked PE/OMMT (XLPE/OMMT) nanocomposites	influence of water-tree aging on the lamellar thickness distribution	**2023**	^108^
Jandaghian et al.	ethylene/1-butene copolymers	influence of Zigler-Natta catalysts on comonomer distribution		^109^
Saleki and Khorshidi	LLDPE	lamellar thickness, short chain branches content (SCBC), and methyl sequence lengths (MSL) on Ziegler–Natta and metallocene catalysts PEs		^110^
Tannous et al.	PET–PE films	SCB distribution		^111^
Liu et al.	PE elastomer	chain structure analysis of PE elastomer fractions	**2024**	^112^
Mansouri et al.	HDPE and HDPE modified with nanosilica	influence of density and nanosilica modification on the chain structure and its relationships with stress cracking resistance		^113^
Long et al.	mLLDPE	molecular structure, methylene sequence distribution, and short-chain branch distribution by TREF × SSA and TREF × HT-GPC cross-fractionation analysis		^114^
He et al.	PE resins for natural gas pipe	chain microstructure of initial resins and fractions		^115^
Chang et al.	PP	influence of piperidine methyl dimethoxysilane (Donor-PMe) on the catalytic activity, isotacticity, molecular weight distribution, isotactic sequence length, and isotactic sequence distribution of PP.		^116^
Su et al.	PP	combined SSA and electrical measurement and the relationship between molecular structure and high-temperature dielectric properties of PP		^117^
Balzer et al.	LDPE blends	branching distribution of LDPE during the deconstruction process		^118^
Sattari et al.	bimodal PE	SSA-based validation of ethylene sequence length distribution models through lamellar thickness and bimodality analysis	**2025**	^119^
Wang et al.	PE elastomer	SSA fractionation of POE crystallizable sequences supporting FSC crystallization kinetics		^120^
Liao et al.	PE-containing block copolymers	influence of confinement on SSA fractionation		^121^
Shao et al.	poly(propylene-*co*-ethylene-*co*-1-butene) terpolymers	sequence lengths, distributions, and formation of the triclinic γ form		^122^
Kurokawa et al.	ethylene/1-hexene copolymers (ternary hybrid catalyst)	short chain branches depending on the ternary hybrid catalyst		^123^
Song et al.	cross-linked PE	influence of cross-linking degree and processing conditions on lamellar thickness and distribution		^124^
Pérez and Satti	*i*PP, *s*PP, and α-olefin copolymers	structural variations in metallocenic PP and its copolymer, including morphological changes induced by gamma irradiation		^125^
***others***				
Colonna et al.	pCBT-RGO nanocomposites	high-temperature peak generated by the supernucleating effect of RGO	**2017**	^126^
Wang et al.	PA1012/PA612 blends	probing the immiscible character of the blends		^127^
Pérez-Camargo et al.	PES–PPS copolymers	influence of chain primary structure and topology (branching) on the crystallization behavior	**2019**	^128^
Fernández-d’Arlas et al.	TPUs	application of SSA on TPUs and enhancement of the crystallinity and WAXS signals through the SSA fractionation	**2021**	^129^
Li et al.	PA1012	competition between chain extension and cross-linking		^130^
Franco-Urquiza et al.	EVOH nanocomposites	influence of the extrusion process on structural modifications		^131^
Fernández-d’Arlas et al.	TPUs	enhancement of the crystallinity and WAXS signals through the SSA fractionation	**2022**	^132^
Gao et al.	TPUs	hard block length distribution		^133^
Maria et al.	P(VDF-*co*-TrFE) copolymers	fractionation capacity of copolymers		^134^
Liu et al.	TPUs	identification of minor differences in hard block length distribution	**2023**	^135^
Wang et al.	TPUs	number and distribution of monomer units in hard blocks		^136^
Schmarsow et al.	PE-*b*-PEO to generate supramolecular networks	influence of the network components on PE and PEO crystallization		^137^
Shang et al.	PEKK copolymers	lamellar thickness distribution		^138^
Han et al.	PEEKs	cross-linking mechanism	**2024**	^139^
Da et al.	EVOH	distribution of ethylene sequences		^140^
Zhang et al.	ethylene-methacrylic acid (EMAA) copolymers	influence of sodium content on lamellar thickness distribution	**2025**	^141^
Zanchi et al.	P(VDF-*co*-TrFE) copolymers	information on Curie transition and ferroelectric crystal populations		^142^

The use of SSA to determine the short-chain branching distribution in ethylene/α-olefin copolymers has recently been the subject of a new ISO standard method published in 2025,^11^ consolidating the technique as a quality control tool in the polyolefin-producing industry. Within this standard, SSA is applied as a thermal fractionation method that resolves polymer chain populations with different short-chain branching contents based on their crystallization and melting behavior, yielding a reproducible fractionation profile suitable for standardized quality assessment.

Three major reviews (2005,[Bibr ref2] 2015,^3^ and 2022 ^4^) have thoroughly consolidated the fundamentals of SSA and its correct application. Building on this foundation, the present review does not aim to rediscuss methodological aspects that are already well established. Instead, a concise overview of key methodological principles and best practices is intentionally retained to keep the manuscript self-contained and to facilitate interpretation of the application discussed herein.

Rather than emphasizing novelty in individual case studies, this review examines the progressive evolution of SSA over nearly three decades, focusing on how its scope, role, and interpretative power have expanded across different material classes. In this context, representative applications are discussed to illustrate the progressive transition of SSA from its original polyolefin focus to increasingly complex and sustainability-driven material systems, including recycled polymers, biobased and biodegradable materials, nanocomposites, and high-throughput calorimetric approaches such as SSA coupled with FSC.

By reframing SSA as a modern tool for material innovation, this review aims to serve as both a practical guide and an updated roadmap for researchers seeking to leverage its full potential in addressing today’s materials science challenges.

## 2 Experimental Foundations of SSA

The SSA technique relies on precise control of a polymer’s self-nucleation (SN) behavior to determine the initial fractionation conditions. Within each SSA sequence, after a first step that only produces self-nucleation (conditioning the sample by maximizing the number of self-nuclei without any fractionation), subsequent SN steps provoke self-nucleation and annealing, thereby producing thermal fractions. In practice, successive SN steps identify the temperature range in which self-nucleation occurs without inducing annealing, thereby defining the safe starting conditions for constructing a meaningful SSA fractionation sequence. Without identifying the ideal self-nucleation temperature (*T* *_s,ideal_*), it is impossible to design a meaningful SSA protocol.

Over nearly three decades of refinement, this combination of SN and controlled annealing has evolved into a standardized framework for probing molecular and lamellar heterogeneity in semicrystalline polymers. The conceptual sequence of both processes is summarized in Scheme 1.

**Scheme 1:**
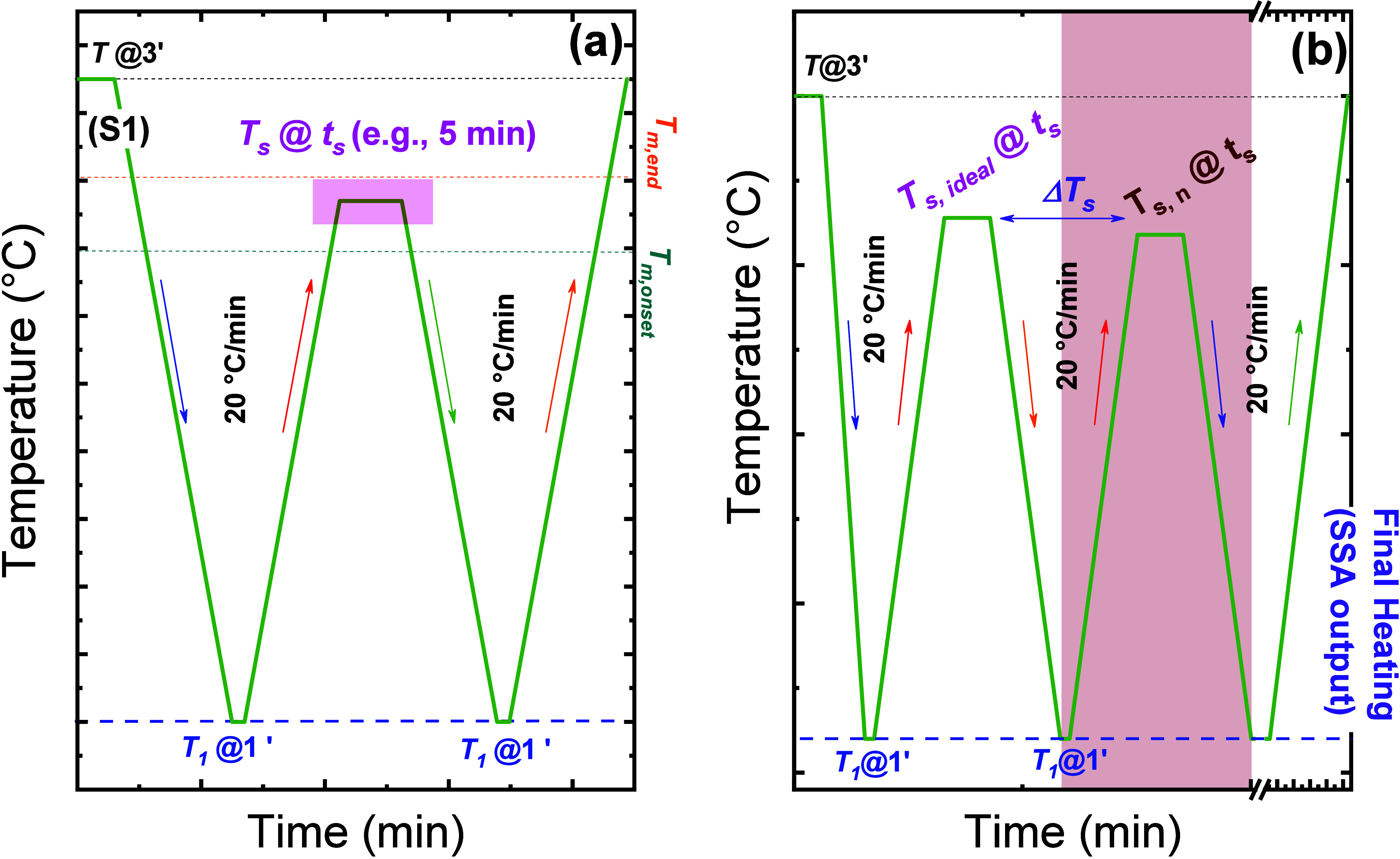
Schematic Representation of (a) Self-nucleation (SN) and (b) Successive Self-nucleation and Annealing (SSA) Protocols^a^

a In both protocols, the sample is first heated to *T* (defined as *T*_*m*_ + 30 °C) to completely erase its previous thermal history. The sample is then cooled to *T*_1_ and held to establish a standard thermal state. In the SSA protocol (b), the self-nucleation step is repeated *n* times at progressively lower temperatures (*T*_*s*_ – Δ*T*_*s*_), schematically denoted as *T*_*s, n*_, before the final heating scan.

In an SN experiment (Scheme 1a), the polymer undergoes controlled melting, self-nucleation, and recrystallization steps. The comparison of cooling (Figure 2a) and heating (Figure 2b) scans before and after holding at *T*_*s*_ allows identification of distinct self-nucleation Domains (Figure 2c). Detailed experimental sequences are extensively described in previous SSA reviews^2–4^ and are not reiterated here.

**Figure 2:**
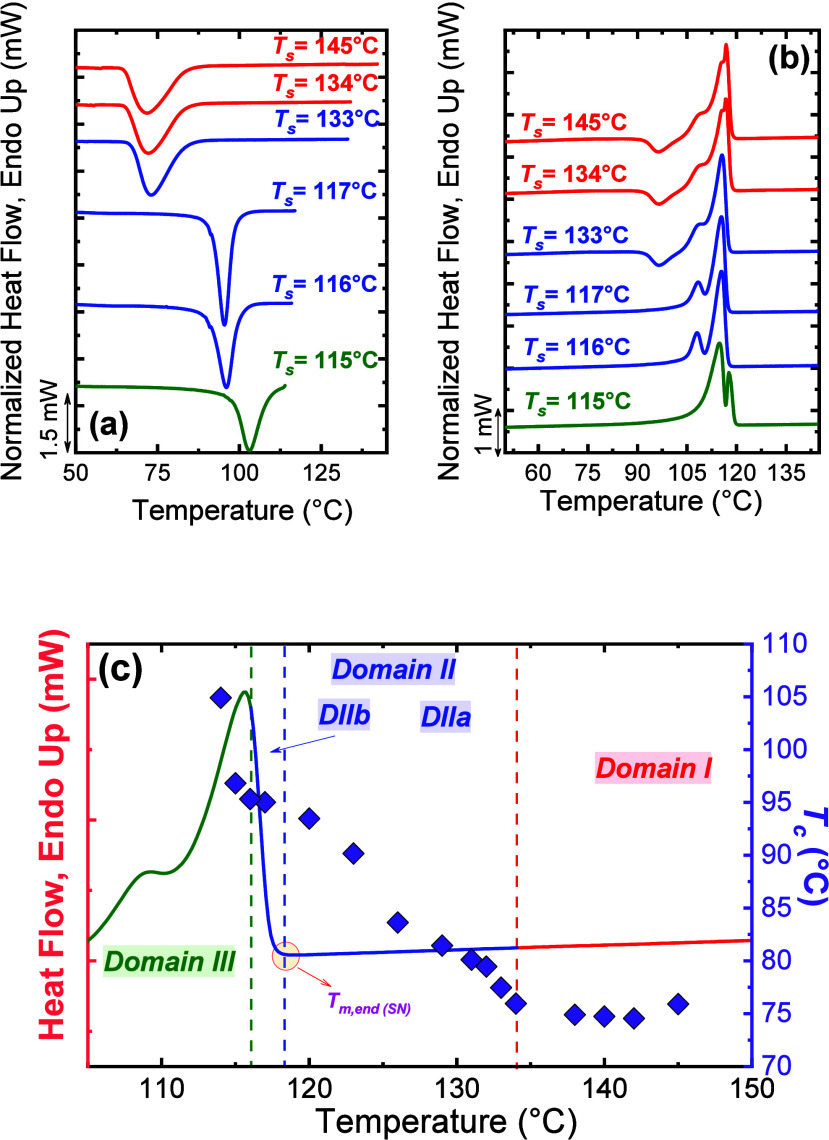
(a) Cooling and (b) heating DSC scans after holding the sample at the indicated *T*_*s*_ values. The curves in *Domain I*, *II,* and *III* are indicated with red, blue, and green colors, respectively. In (c), the standard DSC heating curve (in *Domain I*) is plotted superimposed with the *T*_*c*_ values (right-hand side *y*-axis) vs *T*_*s*_ values (*x*-axis). The obtained *Domains* from (a) and (b) analyses are indicated. Figure 2 is adapted from ref 12. Copyright 2015 American Chemical Society.

Briefly, three SN domains are identified (Figure 2 and Scheme 3):

1. ***Domain I or Complete melting Domain (DI)*:** all crystals melt, yielding an isotropic melt with no memory of its previous crystalline state.
2. ***Domain II or Self-nucleation Domain (DII)*:** small crystal fragments or ordered regions that persist in the melt, acting as self-seeds that raise *T*_*c*_, i.e., self-nucleating the material, without causing annealing. Müller et al. recently divided *Domain II* into two sub-*Domains.* ^13,14^

***Domain IIa or Melt memory Domain (DIIa)*:** Involves complete crystal melting but leaving ordered regions in the melt, where chains “remember” the conformations they had in the crystalline state
***Domain IIb or Self-seeding Domain (DIIb)*:** Involves surviving crystal fragments (seeds) and encompasses the ideal SN temperature *T* *_s,ideal_*.
3. ***Domain III or Self-nucleation and annealing Domain (DIII)*:** Part of the crystalline phase remains unmolten long enough to thicken or reorganize (i.e., annealing), producing additional high-temperature melting peaks.

The influence of *T* *_s_* on crystallization and melting is illustrated in Figure 2, which shows typical DSC cooling and heating scans for poly­(butylene succinate) (PBS). The transition from *Domain I* to *Domain III*, along with the associated shifts in *T*_*c*_ and *T*_*m*_, enables the experimental determination of *T*_*s,ideal*_, located at the lower boundary of *Domain IIb,* where maximum nucleation density is produced without annealing.

While the SN step defines the boundaries of *Domains* within a single SN cycle, the SSA protocol expands this idea into a systematic fractionation sequence. The SSA protocol extends the SN concept into a systematic fractionation sequence by applying consecutive self-nucleation and annealing steps at progressively decreasing temperatures, starting from *T*_*s,ideal*_ (Scheme 1b). The final heating scan yields a characteristic SSA profile in which each melting peak corresponds to a thermally separated population of lamellae with distinct stability and thickness (Figure 3). For detailed experimental protocols, the reader is referred to previous comprehensive SSA reviews.^2–4^

**Figure 3:**
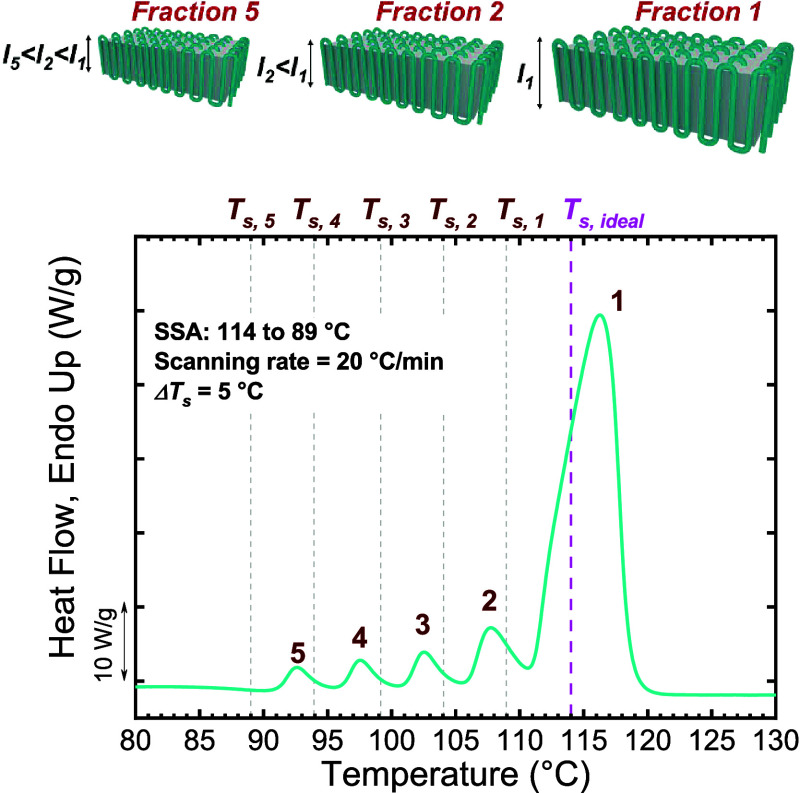
SSA profile of PBS. The vertical lines represent the employed *T*_*s*_, while the generated fractions are labeled. At the top, it is illustrated that the crystals that melt at the highest temperature in Fraction 1 correspond to crystals of thicker lamellar thickness, whereas those crystals that melt at the lowest *T*_*m*_ fractions are thinner crystals. The PBS SSA profile was obtained from ref 15. Figure 3 is adapted from^15^. Copyright 2020 American Chemical Society.

This iterative process performed by SSA yields a fingerprint of the material’s heterogeneity, allowing direct insight into its lamellar stability, thickness distribution, and compositional uniformity. The final heating run of an SSA-fractionated PBS sample is shown in Figure 3. PBS is a linear polymer that, in principle, does not contain defects along the chain, so one would only expect fractionation based on the distribution of molecular weights. However, previous studies on linear polyethylene samples indicate that, although thermal fractions could in principle be obtained, the required fractionation times would be extremely long.

However, recently, Sangroniz et al.^16^ found that intermolecular interactions in polar samples, such as polyesters, can induce SSA fractionation. Indeed, interactions between ester groups normally induce specific conformations in crystals that pair the ester groups of different molecules. These interactions act like physical points that attach chains together, thereby facilitating fractionation based on differences in chain length.^16,17^ SSA was able to generate different fractions, producing a multimelting peak SSA fractionation profile in PBS. The highest-temperature fraction (Fraction 1) corresponds to the thickest, most stable lamellae, while subsequent, lower-temperature peaks reflect progressively thinner lamellae annealed at lower *T* *_s_* values, as shown by the schematic representation of Fractions 1 to 5 in Figure 3.

In the case of linear PBS, the fractionation profile is typical of linear homopolymers without intrinsic defects along the chains (like branches, tacticity differences, comonomer sequences, etc.). The melting peak associated with the highest-temperature fraction is broad and asymmetric, suggesting partial overlap or limited resolution among high-temperature melting populations. In addition, cumulative annealing may have occurred, so the first produced fraction during 5 min at *T*_*s,1*_ could have been further annealed during the 5 min at *T*_*s,2*_.

### 2.1 Best Practices for Variable Selection

Having established the experimental foundations of SN and SSA, the following section summarizes three decades of knowledge into a concise interpretative framework for variable selection, providing practical guidance to enhance resolution, reproducibility, and interpretive power in modern SSA experiments.

The performance and interpretability of an SSA experiment depend critically on a few interconnected experimental variables. As summarized in Scheme 2, four parameters govern the design and outcome of any SSA protocol: the starting temperature (*T* *_s_*), the holding time at *T* *_s_* (*t*_*s*_), the heating and cooling rates, and the fractionation windows (Δ*T*_*s*_) selected for analysis. Their proper balance defines the resolution, reproducibility, and relevance of the results.

**Scheme 2:**
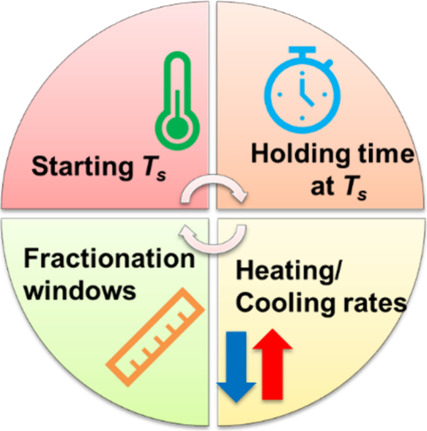
Summary of the Key Variables to Correctly Design an SSA Protocol

#### 2.1.1 Starting Temperature (*T_s_*): The Core of SSA

The selection of *T* *_s_* is the cornerstone of the SSA protocol, as it dictates whether the material undergoes complete melting, self-nucleation, or annealing. The ideal self-nucleation temperature, *T*_*s,ideal*_, is determined experimentally through a preceding SN experiment, which probes the polymer’s response to progressive partial melting.

This concept was first formalized by Fillon et al.,^18^ who identified the three characteristic self-nucleation *Domains* that characterize the outcome of the experimental SN protocol for semicrystalline polymers. Their SN framework, later refined by Müller et al.^3,4,13,19^ remains the foundation for all subsequent SSA developments. Below, we give further details on the SN domains, which are schematically illustrated in Scheme 3.

**Scheme 3:**
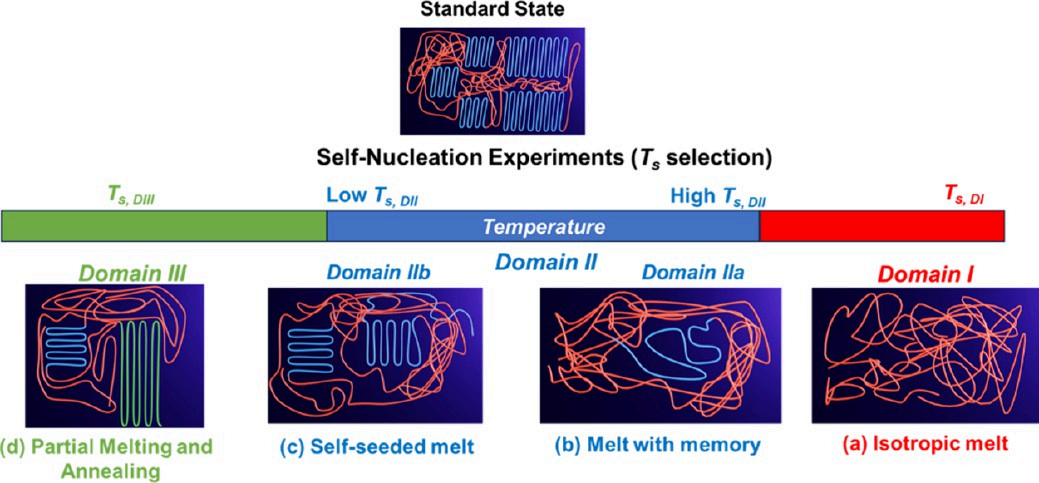
Schematic Representation of the Different Effects of the Holding Time at *T*_*s*_: (a) Isotropic Melt (*Domain I*); (b) Melt with Memory (*Domain IIa*); (c) Self-seeded Melt (*Domain IIb*); and (d) Partial Melting and Annealing (*Domain III*)­[Fn sch3-fn1]

a
Scheme 3 is adapted with permission from ref 3. Copyright 2015 Elsevier.

##### 2.1.1.1 Domain I or Complete Melting Domain (DI)

When the polymer is heated to a temperature well above its melting point (typically 25–30 °C above the *T*_*m*_), the thermal history and melt memory are erased and the melt recovers its isotropic relaxed state, where polymer chains have random coiled conformations (Scheme 3a).

##### 2.1.1.2 Domain II or Self-nucleation Domain (DII)

When *T*_*s*_ is not high enough to reach the isotropic melt, as in *Domain I*, small ordered regions or crystal fragments survive melting and act as self-nuclei during the next cooling step. The crystallization temperature increases relative to *T*_*c*_, evidencing self-nucleation without annealing.

Building on Fillon’s et al.^18^ original definition, Müller et al.^3,4,13,19^ subdivided this *Domain* into two sub-*Domains* that differ in the nature of the self-nuclei produced depending on the *T*_*s*_ range:

##### 2.1.1.3 Domain IIa or Melt with Memory Domain (DIIa)

This region begins when the end of the melting endotherm intersects the baseline of the DSC trace, i.e., when all crystals melt from a calorimetric point of view (as no more endothermic heat flow is recorded). Although no crystalline fragments remain, the melt still retains short-range orientational order (preserved by intermolecular interactions in polar polymers) or transient chain alignment. These ordered regions in the melt, represented by the blue chains in Scheme 3b, are capable of promoting self-nucleation upon cooling. It should be noted that this intersection point does not necessarily coincide with the extrapolated *T* *_m,end_* obtained from the peak analysis, which could slightly underestimate the true onset of *Domain IIa*.

##### 2.1.1.4 Domain IIb or Self-seeding Domain (DIIb)

This sub-*Domain* covers *T*_*s*_ values slightly below the end of the melting range (i.e., lower than the intersection point of the melting endotherm with the baseline). In this *Domain*, small crystal fractions remain unmolten, represented as small lamellar regions (in blue) in Scheme 3c, providing epitaxial self-seeds that act as efficient self-nucleation sites during the subsequent cooling. These self-seeds do not undergo significant reorganization during the holding time, and annealing does not occur.

The *T*_*s ideal*_ is experimentally identified as the lowest *T*_*s*_ within *Domain II*; therefore, it always falls within *Domain IIa*, and corresponds to the highest crystallization temperature (*T*_*c,max*_) observed during the SN experiment within *Domain II*, where the nucleation density reaches its maximum before the onset of annealing.

##### 2.1.1.5 Domain III or Self-nucleation and Annealing Domain (DIII)

At still lower *T*_*s*_ values, only partial melting occurs, leaving a substantial part of the crystalline lamellae unmolten long enough to reorganize or thicken (i.e., they anneal during the 5 min holding period at *T*_*s*_), leading to the appearance of additional high-temperature melting peaks in subsequent DSC heating scans. This is schematically represented as thicker green lamellar crystals in Scheme 3d. *Domain III* thus marks the transition from only self-nucleation (*Domain II*) to self-nucleation and annealing. Self-nucleation still occurs by self-seeding on unmolten and annealed crystals, and *T*_*c*_ values at the onset of *DIII* can increase even further.

The correct identification of *T*_*s,ideal*_, located at the lower boundary of *Domain IIb*, just before the onset of *Domain III*, is essential to ensure that the first step of the SSA protocol induces only self-nucleation without lamellar reorganization.

##### 2.1.1.6 *T_s_* Selection: Quantitative vs Qualitative Protocols

Once *T*_*s, ideal*_ has been experimentally determined, its use within the SSA protocol depends on the nature of the information sought. Two complementary experimental strategies, quantitative and qualitative SSA protocols, have been established to tailor the design of fractionation sequences to the research objective.^3,4^

SSA has demonstrated ***quantitative capability*** in well-defined systems, most notably in polyolefins, where carefully calibrated protocols and cross-validation against complementary techniques (e.g., TREF, NRM, CRYSTAF) have enabled the extraction of meaningful molecular or lamellar distributions. These include, for example, the distribution of short-chain branches, comonomer composition, stereodefects, or cross-link density. In such cases, accurate experimental determination of *T*_*s,ideal*_ for each sample, and strict control of experimental conditions are essential.^3^

Starting from *T*_*s,ideal*_ ensures that the initial SSA step induces self-nucleation only, while subsequent lower *T*_*s*_ values progressively anneal, producing specific lamellar populations or thermal fractions. Because *T*_*s,ideal*_ defines the upper boundary of the fractionation window, any deviation alters the sequence of melting fractions and compromises quantitative comparability.

***Qualitative SSA protocols***, in contrast, are designed to compare a series of samples under identical thermal histories. This is particularly useful for assessing the effects of comonomer type, molecular weight, processing, or degradation in a sample series. A common *T*_*s*_ is selected for all samples, typically the highest *T*_*s,ideal*_ among them (i.e., from the sample with the highest melting point). This choice ensures that none of the samples undergo annealing during the initial SSA step and that any differences in their SSA profiles arise solely from intrinsic structural variations.

In this context, SSA is most robustly applied as a qualitative comparative or ranking tool, enabling the reproducible ordering of samples under identical thermal protocols according to relative differences in crystallizable sequence length, lamellar stability, defect density, or comonomer inclusion, rather than the extraction of absolute distributions.

In certain cases, particularly for highly degradable polymers, e.g., poly­(3-hydroxybutyrate) (P3HB),^20^ some comparative studies have minimized repeated high-temperature erasure steps to reduce the risk of degradation or side reactions.^4^ Similarly, some qualitative studies employ a fixed sequence of decreasing *T*_*s*_ values starting from a high *T*_*s*_ within *Domain II* or even beyond the equilibrium melting temperature (*T*_*m*_°) to facilitate comparison across samples while preserving relative self-nucleation conditions. These approaches should be applied cautiously and explicitly reported, as they depart from standard SSA protocols.

These examples illustrate that *T*_*s*_ is a flexible yet sensitive parameter: while it can be adapted to the system under study, its selection must always be made consciously to ensure meaningful and reproducible comparisons.

Accordingly, the degree of quantification achievable by SSA is system-dependent. In systems where the methodology is rigorously validated (e.g., polyolefins), SSA can provide semiquantitative to quantitative insights, whereas in chemically complex, heterogeneous, or multiphase materials, SSA primarily provides robust comparative trends and fingerprints rather than absolute distributions.

In summary, the proper selection of *T*_*s*_, whether sample-specific or constant for a sample series, defines the analytical scope of the SSA experiment. When correctly chosen, it ensures that the fractionation sequence reflects genuine morphological heterogeneity rather than differences in experimental conditions.

#### 2.1.2 Holding Time at *T_s_*: Balancing Self-nucleation and Annealing

The holding time (*t*_*s*_) or fractionation time defines how long the sample remains at *T*_*s*_ and directly influences the competition between melting, isothermal crystallization, and lamellar thickening. Shorter times (*t*_*s*_ = 1–3 min) are recommended for materials prone to degradation or chain scission, while standard conditions (*t*_*s*_≈ 5 min) allow sufficient self-nucleation for thermal fractionation to be close to completion, depending on the material. In specific cases, longer holds (*t*_*s*_ > 10 min) could induce some recrystallization and annealing, shifting the experiment toward *Domain III* behavior.

The *t*_*s*_ used in the SN experiment for determining *T*_*s,ideal*_ must be identical to that used in the SSA protocol to ensure consistency. Although *t*_*s*_ values between 5 and 15 min generally yield comparable results, shorter holding times are preferred to minimize experimental duration and thermal exposure. In addition, even shorter *t*_*s*_ values are particularly useful for studying early stages of thermal fractionation using FSC.^9,21^

Subminute holding times can capture the kinetics of self-nucleation and annealing in real time, revealing transient phenomena inaccessible by conventional DSC. Thus, *t*_*s*_ is an adaptable parameter that should be tailored to the material’s thermal stability and the specific research objective, whether to map steady-state domain behavior or probe the dynamics of crystal reorganization under rapid heating and cooling.

#### 2.1.3 Heating and Cooling Rates: Controlling Resolution and Kinetics

Thermal scanning rates determine both the degree of supercooling during crystallization and the sharpness of the melting peaks obtained. Moderate rates (10–20 °C/min) typically offer the best compromise between resolution and duration. Faster scans (up to 50 °C/min) are often employed when kinetic information or extensive data sets are required, while FSC allows rates exceeding 1000 °C/s to probe transient crystallization and early stage fractionation phenomena.^9^

For accurate comparison, the same heating and cooling rates must be maintained throughout all SN and SSA cycles, as they influence both the crystallization kinetics and the observed fractionation pattern.

#### 2.1.4 Fractionation Windows: Defining the Experimental Resolution

The temperature intervals chosen for SSA steps define the “fractionation windows” (Δ*T*_*s*_) of the experiment.

Narrower windows (Δ*T*_*s*_ = 2–2.5 °C) are designed to yield narrow, highly resolved thermal fractions, enabling the detection of subtle differences in lamellar thickness distributions or comonomer composition, as demonstrated in some ethylene/α-olefin polyolefins and model random copolymers. Wider windows (Δ*T*_*s*_ *=* 5–10 °C) are more suitable for complex heterogeneous systems such as recycled blends, nanocomposites, or biodegradable copolyesters (e.g., PBS/PCL or PBSA systems), where overfractionation may obscure meaningful trends.

The number and spacing of Δ*T*_*s*_ steps should thus be tailored to the system’s complexity: a few broad steps suffice for homogeneous homopolymers that are difficult to fractionate, whereas dense, fine-grained sequences are preferred when compositional or lamellar size dispersity is high.

In summary, mastering these four variables enables full control over the SSA process, from basic thermal mapping to high-resolution fractionation and structure–property correlation. The interdependence of these parameters, illustrated in Scheme 2, underscores the versatility of SSA: by fine-tuning *T*_*s*_, *t*_*s*_, scanning rates, and Δ*T*_*s*_ in concert, one can transform a standard DSC into a powerful fractionation tool capable of resolving the molecular and lamellar complexity of virtually any semicrystalline polymer.

## 3 SSA Applications in the Past Decade: Growing Applications in Sustainable Materials

To map the expansion of SSA over the past decade, Table 1 compiles publications from 2015 to 2025 that explicitly use SSA as a structural, topological, or compositional characterization tool. For clarity, the works are grouped into three categories: (i) sustainable materials, including biodegradable polymers, recycled plastics, and nanocomposites with circular-economy relevance; (ii) polyolefins, historically the core domain of SSA; and (iii) other semicrystalline polymers (e.g., polyurethanes, polyamides, among others) where SSA has more recently emerged as a high-resolution probe of lamellar organization. This classification highlights the steady diversification of SSA beyond its traditional polyolefin scope and its growing relevance to sustainable polymer design.

In addition, for space reasons, each entry in Table 1 is intentionally limited to a concise description of the primary SSA-related contribution of the referenced work, rather than a comprehensive summary of all aspects addressed in the original study. Emphasis is placed on how SSA was used to extract structural, kinetic, or morphological insight beyond conventional DSC, even when the original work also included broader modeling, compositional, or application-oriented analyses. A detailed discussion and critical interpretation of selected representative studies are provided in the subsections below.

In the following sections, we highlight representative case studies from each nonpolyolefin category. These examples were selected not only for their scientific relevance but also for showcasing how SSA has become a high-resolution tool capable of addressing questions arising in modern sustainable materials: from the design of biodegradable copolymers and circular-economy blends to the study of copolymers, and advanced thermoplastic polyurethanes.

### 3.1 SSA on Random and Block Copolymers

#### 3.1.1 SSA of Random Copolymers: Revealing Isodimorphism, Isomorphism, and Mixed Crystallization Modes

SSA experiments have proven to be a powerful tool to elucidate the crystallization mode in random copolymers. Their combination of successive nonisothermal and isothermal steps makes them uniquely sensitive to subtle differences in comonomer inclusion or exclusion, enabling direct differentiation between isomorphic, isodimorphic, and mixed crystallization modes. In addition, the SSA sequence can be used as a controlled crystallization condition or as an alternative method to roughly estimate the equilibrium melting temperature (*T*_*m*_°).

Random copolymers can crystallize following three main modes:^143–146^ isomorphism, isodimorphism, and comonomer exclusion, or even through mixed modes^143,147–149^ where multiple mechanisms coexist across composition. These include combinations such as isodimorphism/isomorphism,^147,148^ comonomer exclusion/isodimorphism,^149^ or even triple mixed modes (comonomer exclusion/isomorphism/isodimorphism).^150^ The balance between comonomer exclusion and inclusion within the crystal lattice determines the particular mode.

This section intentionally focuses on recent and representative SSA studies that have been instrumental in establishing and refining the understanding of crystallization modes in random copolymers, particularly isodimorphism and related mixed behaviors, rather than aiming to provide an exhaustive survey of all copolymer systems reported in the literature.

Scheme 4 summarizes the theoretical behavior of the main crystallization modes, showing the expected evolution of melting (or crystallization) temperature with comonomer content and their corresponding crystalline structures.

**Scheme 4:**
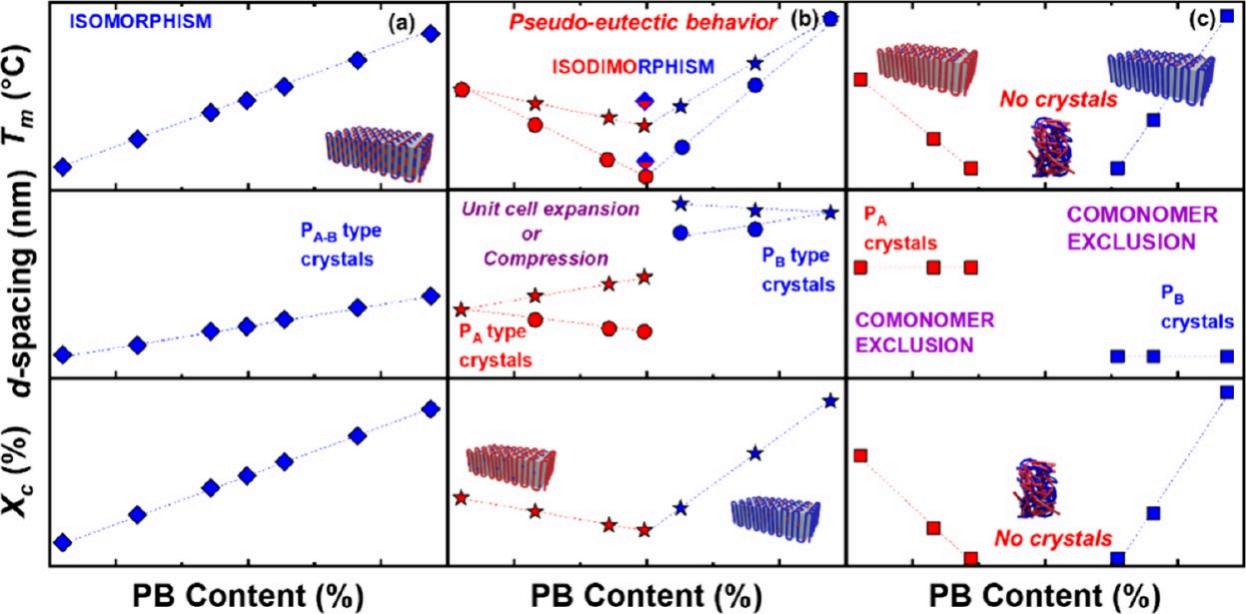
Schematic Representation of Variations in *T*_*m*_ (Top Panel), *d*-Spacing (Middle Panel), and *X*_*c*_ (Bottom Panel) vs PB Content for the Main Crystallization Modes in Random Copolymers (PA*_*x*_* B*_*y*_* Model Copolymer): (a) Isomorphism, (b) Isodimorphism, and (c) Comonomer Exclusion^a^

a In all cases, cartoons represent the crystals with red for comonomer A and blue for comonomer B. The spheres represented the included B or A comonomer in the dominant A or B crystals. Note that, for the comonomer-exclusion case, the comonomer is completely excluded from the crystal lattice of the second comonomer and vice versa.

In an isomorphic copolymer, both comonomers cocrystallize within a single lattice, producing a linear increase in *T*_*m*_ with composition and a single unit-cell type (top of Scheme 4a). Complete cocrystallization occurs only when the repeat units are geometrically and energetically compatible, as reviewed by Pan and Inoue^145^ and Zheng and Pan.^146^ At the opposite extreme, comonomer exclusion results in the progressive suppression of crystallization as one component’s chains disrupt the other’s lattice (bottom of Scheme 4c).

Between these extremes lies isodimorphism (Scheme 4b), in which inclusion and exclusion coexist. At A-rich compositions, A-type crystals incorporate a limited amount of B units, while at B-rich compositions, the opposite occurs. The crossover defines the pseudoeutectic composition at which both crystal forms can coexist.^143–146^

##### 3.1.1.1 SSA in Isodimorphic Random Copolymers

SSA fractionation provides a direct link between the comonomer distribution and the lamellar population in isodimorphic copolymers. Compared to their homopolymer counterparts, isodimorphic random copolymers exhibit more numerous, better-resolved fractions due to interruptions caused by excluded comonomer units. Near or at the pseudoeutectic composition, bimodal SSA profiles typically emerge, reflecting the coexistence of two crystalline phases formed during the applied crystallization conditions and their sequential melting during the SSA heating step. A representative example is poly­(butylene succinate-*ran*-butylene azelate) (BS*_*x*_* BAz*_*y*_*), studied by Arandia et al.,^24^ as shown in Figures 4a,b. Using a common starting temperature *T*_*s,ideal*_ = 116 °C (from PBS) and Δ*T*_*s*_ = 5 °C, SSA revealed that the copolymers fractionate far more effectively than the homopolymers. In PBS and PBAz, fractionation arises mainly from chain-length differences and intermolecular interactions,^16^ whereas in the copolymers, it is dominated by the degree of comonomer exclusion. A similar behavior was also observed by Pérez-Camargo et al.^15^ in PBS-*ran*-butylene adipate (BS*_*x*_* BA*_*y*_*) copolymers (Figure 4c).

**Figure 4:**
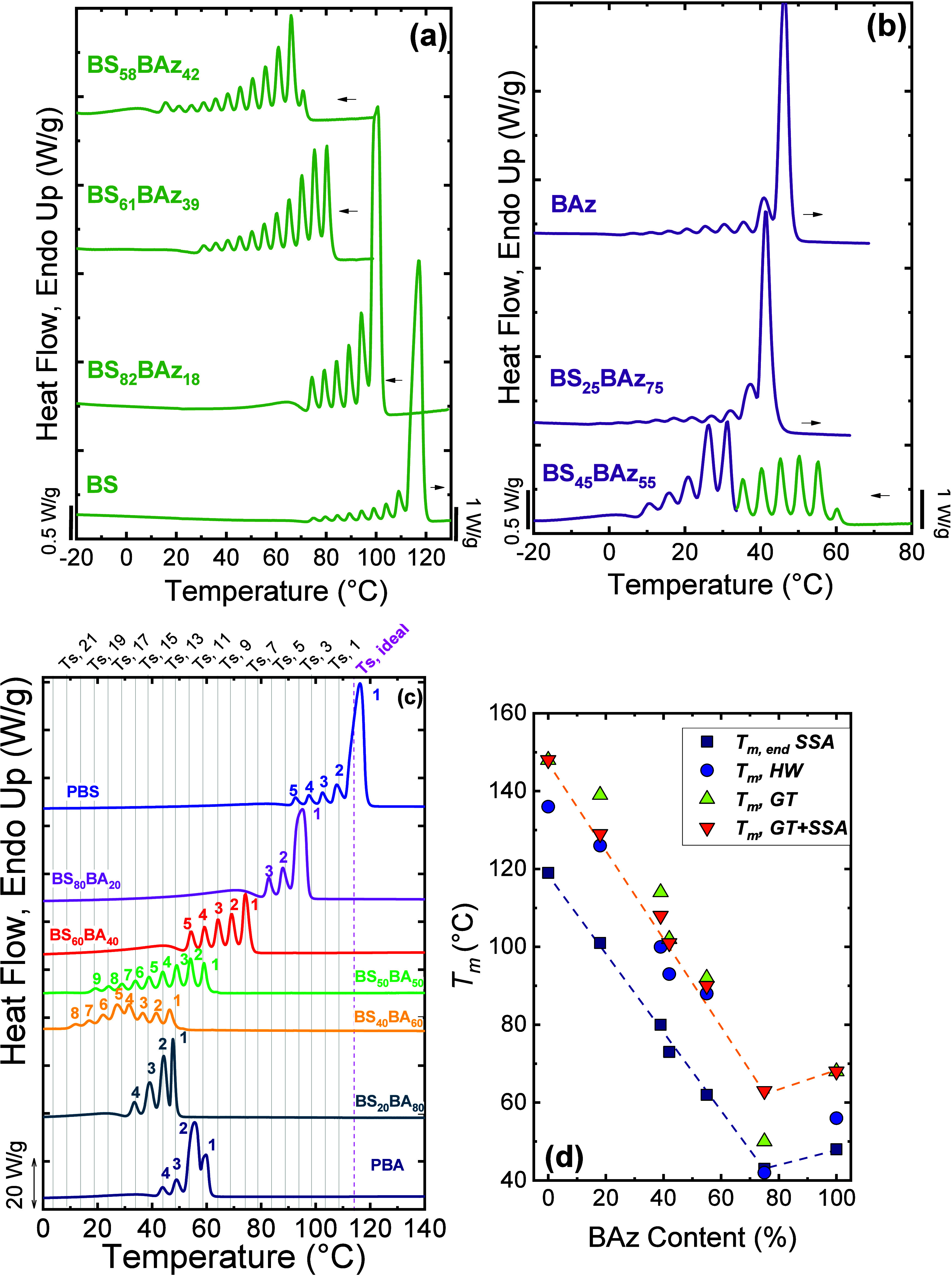
SSA profiles for (a) neat PBS and BS-rich compositions; (b) neat PBAz and BAz-rich compositions; and (c) BS_*x*_BA_*y*_ copolymers. (d) Experimentally obtained end melting temperatures by SSA (*T*_*m,end*_SSA) and equilibrium melting temperatures, *T*_*m*_° (obtained through Hoffman–Weeks (*T*_*m*_ *,* HW), Gibbs–Thomson (*T*_*m*_ *,* GT) and *T*_*m*_ *,* GT + SSA (*T*_*m*_ *,* GT + *T*_*m,end*_SSA, see explanation in the text) versus BAz molar content for PBS, PBAz, and BS_*x*_BAz_*y*_ copolymers. (a) and (b) are adapted with permission from ref 24. Copyright 2016 John Wiley and Sons. (c) is adapted from ref 15. Copyright 2020 American Chemical Society. (d) is adapted with permission from ref 27. Copyright 2019 Elsevier.

As the BAz content increased, the *T*_*m,SSA*_ progressively decreased, consistent with shorter crystallizable sequences. Moreover, the relative intensity of the high-temperature fraction decreased, while the lower-temperature fractions increased in importance (Figure 4a,b), indicating that comonomer content modulates lamellar structure. At the pseudoeutectic composition (BS_45_BAz_55_), a bimodal SSA profile was observed (Figure 4b), with high-*T*_*m*_ fractions corresponding to BS-rich crystals (fractions 1 to 6) and low-*T*_*m*_ ones to BAz-rich crystals (fractions 7 to 11). This bimodal fractionation reflects the coexistence of two distinct crystalline phases and their sequential melting during the SSA heating step. Complementary WAXS/SAXS analyses confirmed the simultaneous presence of both crystalline lattices and demonstrated that the isodimorphic character of the system is largely independent of kinetic effects under the explored crystallization conditions.^24^ Figure 4c shows such a bimodal SSA profile in the BS_40_BA_60_, with fractions 1 to 3 belonging to BS-type crystals and 4 to 8 to BA-type crystals. Interestingly, for the BS*_*x*_* BA*_*y*_* copolymers, there is a significant influence of the crystallization conditions, since under nonisothermal conditions, the sequential melting was also observed for BS_50_BA_50_. However, under SSA conditions, the BS_50_BA_50_ copolymer is dominated by BS-type crystals, accounting for the absence of a bimodal SSA profile. This was one of the first reports of the influence of crystallization conditions, as shown by Pérez-Camargo et al.,^15^ who showed that the inclusion/exclusion balance depends on crystallization conditions: nonisothermal crystallization promotes higher inclusion, isothermal conditions promote stronger exclusion/lower inclusion, while SSA represents an intermediate regime combining both effects. Thus, SSA not only reveals the existing crystallization mode but also enables tuning it through the applied thermal sequence.

##### 3.1.1.2 SSA Reclassifying “Isomorphic” Systems

As shown in Scheme 4a, an isomorphic copolymer should exhibit a linear increase of *T*_*m*_ with composition and a single, compositionally homogeneous crystalline lattice. Yet, these two criteria alone can be misleading. Apparent linearity in *T*_*m*_ vs composition plots and single diffraction patterns may conceal dual crystallization behavior that remains unresolved under conventional thermal analysis.

Zhang et al.^31^ re-examined the crystallization of poly­(hexamethylene carbonate-*co*-hexamethylene urethane) (PHC*_*x*_* U*_*y*_*) block copolymers, previously classified as isomorphic.^151^ Conventional DSC revealed single, linearly shifting *T*_*m*_ and *T*_*c*_ values (Figure 5a,b), and WAXS showed a single crystalline lattice corresponding to the HU phase (Figure 5c).

**Figure 5:**
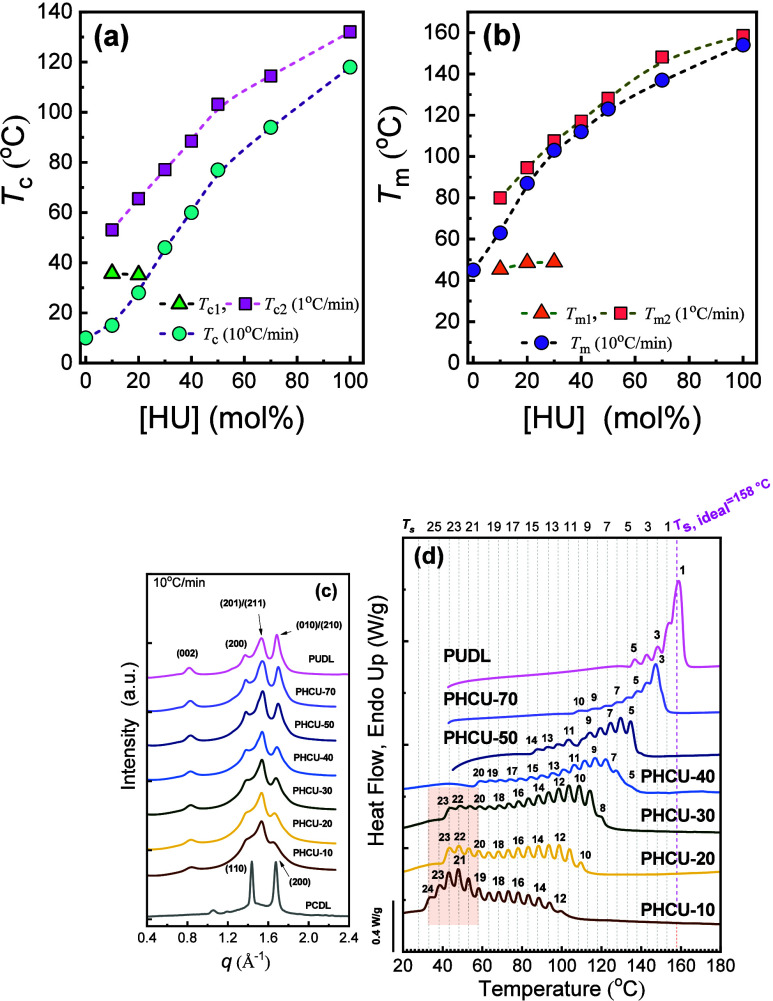
(a) *T*_*c*_ and (b) *T*_*m*_ values versus [HU] content for PHCU copolymers. The scanning rates were 1 and 10 °C/min; (c) 1D WAXS profile of PHCU copolymers cooled from the melt at 10 °C/min and then measured at RT. The WAXS pattern of the PCDL was taken from ref 151. (d) DSC heating scans for SSA-fractionated PHCU copolymers and PUDL oligomer at 10 °C/min. The shadowed region indicates the PC fractions. Figure 5 is adapted with permission from ref 31. Copyright 2021 Elsevier.

To probe deeper, the authors applied SSA with Δ*T*_*s*_ = 5 °C, *t*_*s*_ = 5 min, a scanning rate of 10 °C/min, and a common *T*_*s,ideal*_ = 158 °C (from PU). As shown in Figure 5d, the number and sharpness of SSA fractions increased progressively as HU content decreased. When HU ≤ 40%, a bimodal SSA profile appeared, with low-temperature fractions corresponding to HC-rich crystals. This unexpected bimodality contradicted the earlier classification (as isomorphic copolymer) and revealed a more complex crystallization behavior, where both hard and soft segments crystallize in partially segregated lamellar domains. SSA thus demonstrated that PHC*_*x*_* U*_*y*_* copolymers are neither fully isomorphic nor isodimorphic, they exhibit coexisting lamellar populations whose relative stability depends on block composition.

A similar situation was observed more recently in poly­(ε-caprolactone-*ran*-ω-pentadecalactone) (PCL*_*x*_* PPDL*_*y*_*) random copolyesters.^46^ This series was initially described as isomorphic based on conventional DSC and WAXS, which showed single *T*_*m*_ and *T*_*c*_ values that changed linearly with composition. However, SSA fractionation revealed dual melting domains and a composition-dependent pseudoeutectic behavior, features characteristic of isodimorphism. These findings reclassified the PCL*_*x*_* PPDL*_*y*_* system as isodimorphic, underscoring the diagnostic power of SSA to reveal hidden dual-phase crystallization and correct misinterpretations arising from conventional thermal analyses.

The PCL*_*x*_* PPDL*_*y*_* study reinforced a trend previously seen in the PHC*_*x*_* U*_*y*_* copolymers: even in systems with continuous *T* *_m_*-composition dependencies, SSA can expose the coexistence of independent lamellar populations.

Comparable complexity has also been reported in olefin multiblock copolymers (OMBCs), where topological confinement and partial miscibility between hard and soft segments distort the SSA profiles.^100^ Together, these systems highlight that the apparent simplicity of a single DSC peak or a uniform WAXS pattern can mask intricate microphase segregation and crystallization competition, which only SSA fractionation can resolve.

To date, SSA has not been applied to any confirmed isomorphic random copolymer, and such systems remain an open frontier. However, insights can be drawn from related model materials. For instance, Appiah et al.^62^ used SSA to study precision polyethylene derivatives containing regularly spaced azobenzene defects (H-azo and F-azo) introduced via ADMET polymerization. Using *t*_*s*_ = 5 min, Δ*T*_*s*_ = 10 °C, and *T*_*s*_ = 154 °C, they found no significant fractionation, indicating full inclusion of the defects within the lamellae. Interestingly, *T*_*m,SSA*_ increased slightly relative to the neat polymer, confirming that the defects were accommodated within the crystalline lattice. Analogously, a truly isomorphic copolymer would be expected to show a similar SSA response, minimal fractionation, stable single-domain melting, and *T*_*m,SSA*_ values comparable to those of the parent homopolymers.

Nonetheless, such behavior has yet to be experimentally demonstrated. Instead, most copolymers previously labeled as isomorphic reveal, under SSA, bimodal or multimodal fractionation signatures characteristic of partial counit exclusion. This recurring pattern suggests that the boundary between isomorphism and isodimorphism is not discrete but continuous, shaped by composition, chain topology, and thermal history. SSA, therefore, remains the most sensitive method to map this continuum, capable not only of diagnosing subtle structural dualities but also of redefining how crystallization modes are understood in semicrystalline copolymers.

##### 3.1.1.3 SSA as an Intermediate Crystallization Condition: Bridging Non-isothermal and Isothermal Regimes

Beyond its role as a diagnostic tool, SSA can also be understood as a controlled crystallization environment that bridges nonisothermal and isothermal regimes. Each SSA cycle combines heating-holding-cooling steps, creating a sequence where kinetic and thermodynamic factors alternate in dominance. This cyclical nature positions SSA as an intermediate crystallization condition, enabling the formation of lamellar structures that are neither purely kinetically trapped nor fully equilibrium-driven.

In conventional nonisothermal crystallization, rapid cooling promotes comonomer inclusion as there is not enough time for the saturation of the comonomer exclusion process within the establishment of a particular exclusion/inclusion balance, often leading to thinner lamellae and greater lattice disorder. Conversely, isothermal crystallization allows sufficient chain mobility for comonomer exclusion and lamellar thickening, favoring thermodynamic over kinetic control.

By alternating short isothermal holds at specific *T*_*s*_ with controlled cooling and reheating segments, SSA combines both effects in a balanced manner. It partially melts and recrystallizes the sample in successive cycles, maintaining residual order that promotes self-nucleation while simultaneously allowing limited reorganization during each annealing step. A clear demonstration of this hybrid behavior was provided by Pérez-Camargo et al.^15^ in BS*_*x*_* BA*_*y*_* copolymers. The authors compared nonisothermal, isothermal, and SSA-induced crystallizations, revealing that (a) nonisothermal crystallization led to stronger comonomer inclusion within PBS-rich lamellae, (b) isothermal conditions favored exclusion and lamellar thickening, and (c) SSA produced an intermediate state, with balanced inclusion/exclusion ratios and intermediate lamellar thicknesses. This behavior is clearly observed in Figure 6, where the normalized *d*-spacing for each plane is plotted as a function of BA content for the three crystallization conditions. The nonisothermal conditions exhibit greater variations and greater distortion of the unit cell than the isothermal conditions, leaving the SSA as an intermediate condition, particularly for the PBS-rich compositions (Figure 6a,c). In compositions rich in BA content (Figure 6b,d), the changes are driven by PBA polymorphism, a feature also regulated by SSA.

**Figure 6:**
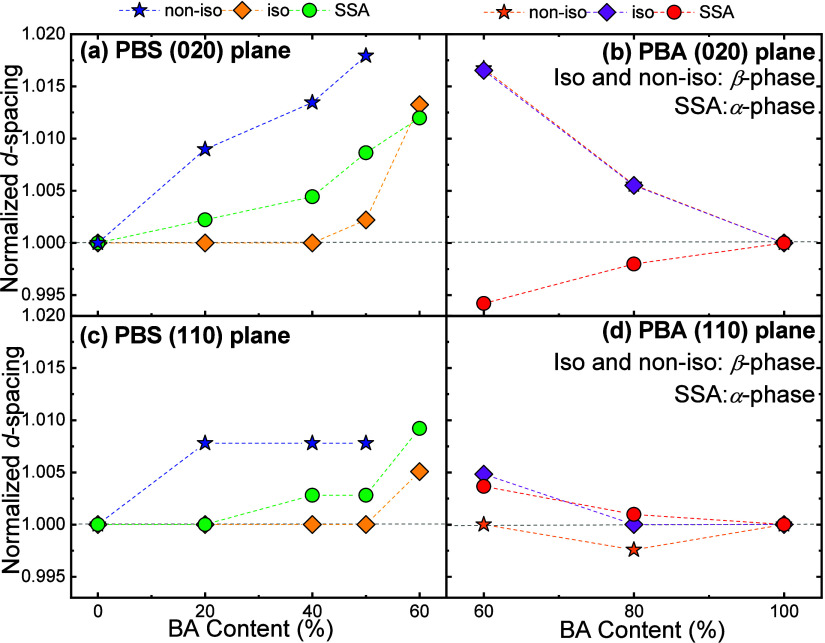
Normalized *d*-spacings for different crystallization conditions (nonisothermal, isothermal, and SSA conditions) as a function of BA content. (a) PBS (020) plane, (b) PBA (110) plane, (c) PBS (110) plane, and (d) PBA (020) plane. Figure 6 is adapted from ref 15. Copyright 2020 American Chemical Society.

Most strikingly, SSA also altered the polymorphism of the PBA-rich phase. While both nonisothermal (Figure 7a) and isothermal (Figure 7b) crystallizations yielded the metastable β-form of PBA-rich copolymers, the SSA protocol selectively promoted the formation of the thermodynamically stable α-form (Figure 7c), even within the copolymers. This β→α transformation occurs via partial melting of the β-form, followed by recrystallization during successive SSA cycles, confirming that repeated self-nucleation and annealing steps can modulate the material’s crystalline phase. This unique ability to drive controlled polymorphic transitions highlights the dual kinetic-thermodynamic nature of SSA and its sensitivity to subtle energetic differences between competing crystalline forms.

**Figure 7:**
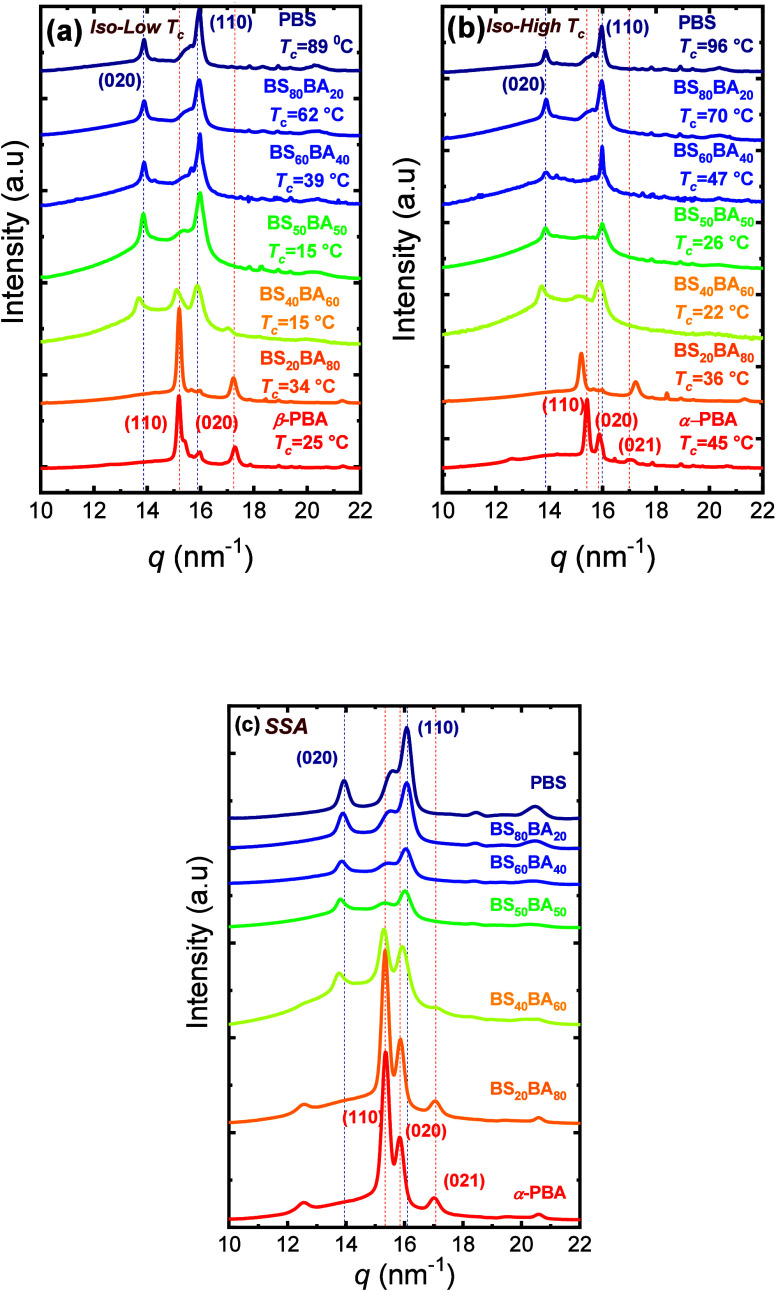
WAXS patterns for PBS, PBA, and BS_*x*_BA_*y*_ copolymers at the indicated (a) low and (b) high *T*_*c*_ values and (c) after SSA fractionation. Note that the WAXS pattern of the BS_40_BA_60_ sample strongly depends on the *T*_*c*_ used (coexistence of BS- and BA-type phase vs BS-type phase only). Dotted lines are used to indicate the main reflections of the PBS and PBA homopolymers. Figure 7 is adapted from ref 15. Copyright 2020 American Chemical Society.

A similar intermediate behavior was reported for PCL*_*x*_* PPDL*_*y*_* copolyesters,^46^ where SSA revealed persistent isodimorphic character independent of cooling rate, confirming that the inclusion-exclusion balance defining the crystallization mode remains stable under SSA cycling. Together, these findings underscore the versatility of SSA: it not only bridges kinetic and thermodynamic control but can also redirect polymorphic selection pathways, allowing the exploration of metastable structures inaccessible under conventional crystallization regimes.

By occupying this unique intermediate regime, SSA emerges not only as a fractionation technique but also as a structure-controlling crystallization route, capable of regulating lamellar purity, comonomer distribution, and crystalline form. This dual analytical-synthetic nature strengthens SSA’s position as a cornerstone method for sustainable polymer design, revealing otherwise hidden morphologies that directly influence mechanical properties, recyclability, and biodegradability.

##### 3.1.1.4 Equilibrium Melting Temperature (*T*_m_°): An SSA-Based Alternative

One of the most practical applications of SSA is its ability to refine crystalline morphology and produce well-defined distributions of lamellar thickness. These refined crystals can be used to approximately estimate the *T*_*m*_° of semicrystalline polymers and copolymers, an essential parameter that describes their thermodynamic stability and comonomer inclusion capacity.

Traditionally, *T*_*m*_° is determined using the Hoffman–Weeks (HW)^152^ or Gibbs–Thomson^153^ (GT) extrapolations, which rely on a series of isothermal crystallizations. However, in random copolymers, these methods often yield scattered or inconsistent results, especially near the pseudoeutectic compositions where dual crystallization occurs. The coexistence of multiple lamellar populations and the difficulty of obtaining steady-state conditions introduce both experimental and extrapolation uncertainties.

To overcome these limitations, Arandia et al.^27^ proposed an innovative SSA-based alternative to estimate *T*_*m*_°, using the end melting temperature after SSA fractionation (*T*_*m,end,SSA*_) as a proxy for the melting of the thickest lamellae generated through successive annealing.

After SSA treatment, the lamellar stacks are progressively thickened and stabilized, approaching thermodynamic equilibrium. The authors corrected the experimental *T*_*m,end,SSA*_ values by adding a constant offset calibrated from the reliable *T*_*m*_°(GT) values of the parent homopolymers, 148 °C for PBS and 68 °C for PBAz. The corresponding offsets were 29 °C for the BS-rich copolymers and 20 °C for the BAz-rich ones, yielding the adjusted values *T*_*m*_° (SSA/GT). This modified approach produced a smooth and physically meaningful trend of *T*_*m*_° versus composition (Figure 4d).

Remarkably, the *T*_*m*_° (SSA/GT) data for the copolymers lie between the classical HW and GT extrapolations, validating the method’s internal consistency while reducing the typical scatter seen in conventional analyses. More importantly, these values enabled a reliable application of the comonomer inclusion/exclusion models of Flory,^154^ Baur,^155^ Sanchez and Eby,^156^ and Wendling and Suter.^157^ From these fits, Arandia et al.^27^ concluded that In BS-rich copolymers, only a minor inclusion of BAz units occurs within the PBS crystalline lattice. Conversely, in BAz-rich copolymers, a more significant incorporation of BS units into the PBAz crystals is possible.

The use of *T*_*m*_° (SSA/GT) was essential to reach these conclusions, as it provided a thermodynamically consistent framework for connecting fractionation behavior with molecular-level inclusion/exclusion mechanisms. This approach highlights SSA’s dual power, not only as a diagnostic tool to reveal structural heterogeneity, but also as a quantitative method to derive equilibrium parameters directly from controlled thermal fractionation, bypassing the limitations of traditional isothermal extrapolations.

#### 3.1.2 Triblock Terpolymers

The remarkable molecular segregation capacity of SSA has proven essential to unravel the complex crystallization behavior of multiblock systems. In triple-crystalline triblock terpolymers, where each block can crystallize independently but under mutual confinement, conventional DSC analysis often fails to disentangle their overlapping thermal signals.

The work of Palacios et al.^29^ on poly­(ethylene oxide)-*b*-poly­(ε-caprolactone)-*b*-poly­(l-lactide) (PEO-*b*-PCL-*b*-PLLA) elegantly demonstrated that SSA can overcome this limitation and reveal the sequential crystallization hierarchy of such systems. After melt crystallization at a slow cooling rate (1 °C/min), standard DSC heating scans (20 °C/min) showed three broad endothermic regions corresponding to the melting of the PLLA, PCL, and PEO blocks at approximately 120 °C, 50–60 °C, and 40 °C, respectively. However, due to partial overlap in the transition regions of PEO and PCL, it was impossible to separate their individual contributions unambiguously.

To address this, Palacios et al. implemented an SSA protocol using the *T*_*s,ideal*_ of the highest-melting block (PLLA, 143 °C), a holding time of *t*_*s*_ = 5 min, Δ*T*_*s*_ = 5 °C, and scanning rates of 20 °C/min. The resulting fractionation profiles are shown in Figure 8.

**Figure 8:**
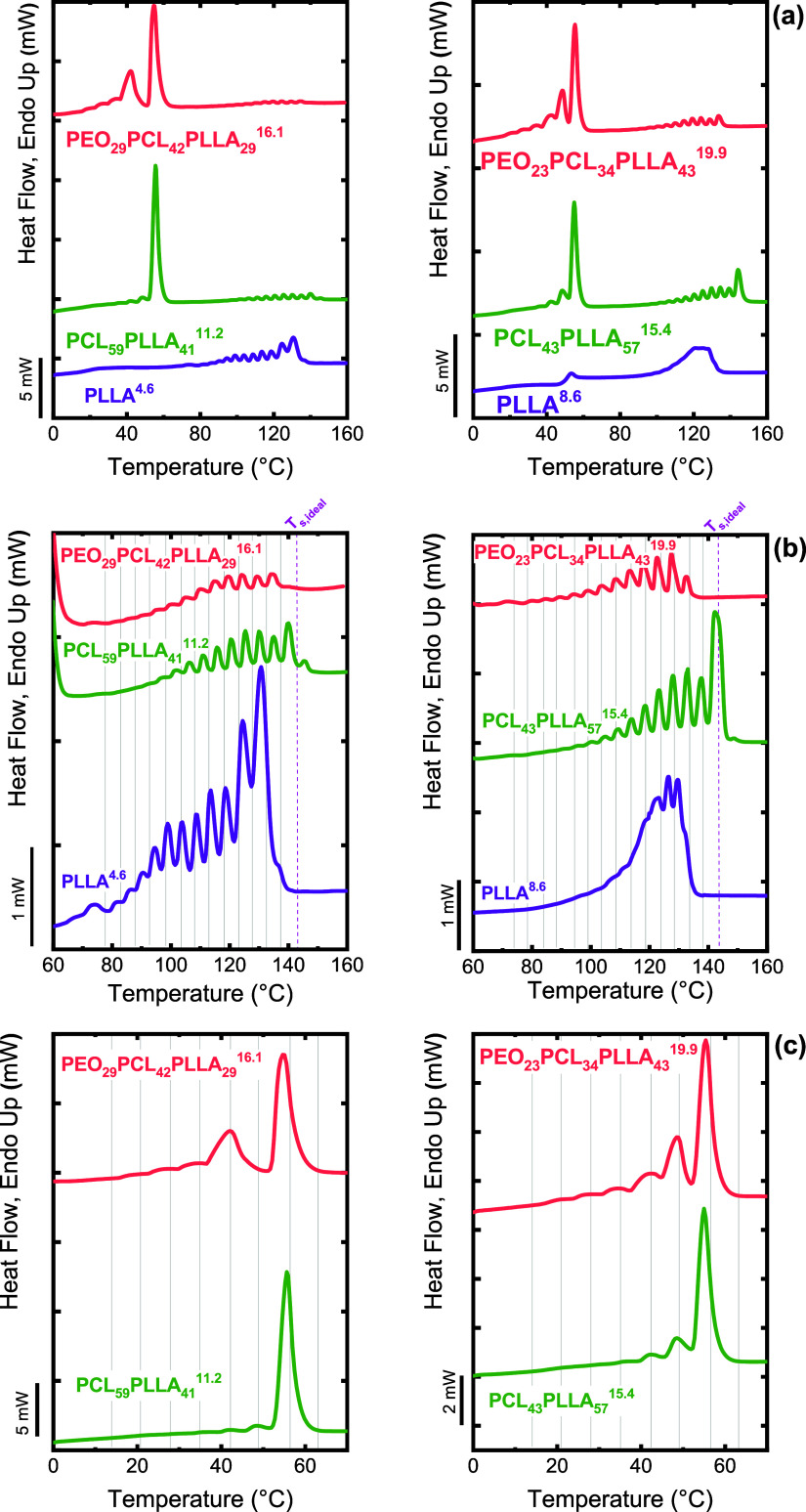
(a) SSA profiles for PEO-*b*-PCL-*b*-PLLA triblock terpolymers, PCL-*b*-PLLA diblock copolymers, and PLLA homopolymer; (b) zoom of the PLLA fractionated zone; and (c) zoom of the PEO-PCL and PCL fractionated zone. Figure 8 is adapted with permission from ref 29. Copyright 2019 John Wiley and Sons.

The SSA profiles clearly revealed that all three blocks can be independently fractionated. For the PLLA block, fractionation was more pronounced at higher PLLA content, confirming its dominant role in the crystallization sequence. Interestingly, the melting temperatures of the PLLA fractions (*T*_*m,SSA*_) were higher in the copolymers than in the neat PLLA, indicating that the presence of molten PCL and PEO chains enhances PLLA’s crystallizability, most likely by providing additional molecular mobility during self-nucleation.

In the lower-temperature region (40–60 °C), SSA successfully resolved the individual melting contributions of PCL and PEO. The peaks above 50 °C corresponded to the melting of PCL lamellae, while those below 45 °C originated from PEO crystals.

In situ WAXS measurements during the final SSA heating corroborated this interpretation: the diffraction peaks of PEO disappeared first, followed by those of PCL, confirming the sequential melting order inferred from DSC. Through this combined DSC/SSA-WAXS analysis, Palacios et al.^29^ conclusively demonstrated that SSA can effectively deconvolute the crystallization behavior of highly complex multiblock systems.

In the PEO-*b*-PCL-*b*-PLLA terpolymer, the SSA technique not only distinguished the contributions of each block but also revealed the hierarchical crystallization order, i.e., PLLA → PCL → PEO, underlying the formation of triple-crystalline architectures. This work stands as a benchmark example of how SSA enables access to a level of morphological and thermodynamic detail that remains hidden to conventional thermal techniques, even in the most compositionally intricate polymer systems.

### 3.2 Topology Effects on SSA Fractionation

#### 3.2.1 Topology Effects: Cyclic vs Linear Topology

The influence of molecular topology on SSA fractionation provides key insight into how chain architecture governs crystal thickening and diffusion. The comparison between cyclic and linear architectures is particularly revealing because both share chemical composition and molecular weight, yet differ in topology. The influence of chain topology (cyclic vs linear) in the SSA fractionation was illustrated in our previous works,^3,4,158^ by fractionating cyclic and linear PCLs of equal MW. By applying the same SSA protocol, it was found that, independently of MW, the *c*-PCL possesses a greater annealing capacity, forming thicker lamellae that melt at a higher *T*_*m*_ than its analogous *l*-PCL. Note that thermodynamically, *l*-PCL can be extended to twice the maximum length of *c*-PCL. Therefore, the remarkably higher annealing capacity of the *c*-PCL is explained by the kinetic factors that dominate the SSA experiments. During the 5 min annealing time, the *c*-PCL exhibits higher mobility and a reduced entanglement density that facilitates lamellar reorganization and diffusion.^3,4^

SSA has also proven to be an efficient tool to discriminate topological effects in other homopolymers, such as cyclic and linear poly­(l-lactide) (PLLA) and poly­(d-lactide) (PDLA).^26^ Zaldua et al.^26^ evaluated both topological and stereochemical differences, preparing cyclic polymers via ring-closure “click” chemistry and linear counterparts by ring-opening polymerization. All samples (*M*_*n*_ ≈ 14,000–16 700 g/mol) were characterized under identical SSA conditions (*T*_*s, ideal*_ = 155.5 °C, Δ*T*_*s*_ = 5 °C, 10 cycles).

Figure 9a displays the final heating traces after the SSA procedure. The cyclic PLLA and PDLA exhibit a higher annealing capacity than their linear analogues, reaching Fraction 1, whereas linear polymers only fractionate up to Fraction 2. These differences arise from the lower entanglement density of cyclic chains, which promotes lamellar thickening within the fixed annealing time.

**Figure 9:**
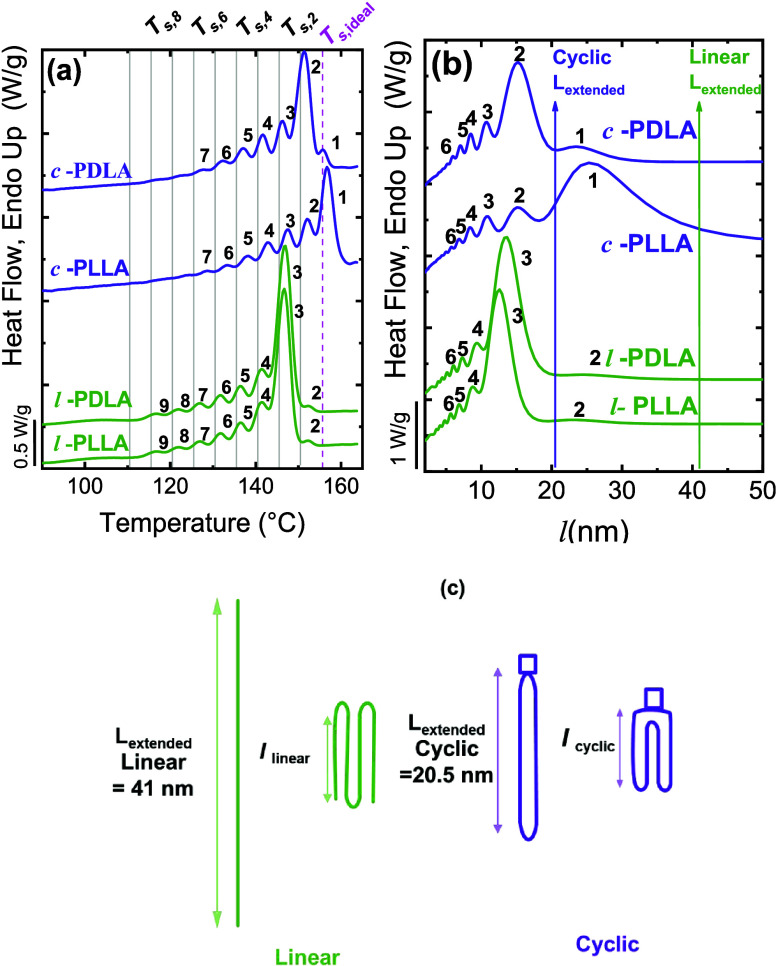
SSA profiles for all the samples as a function of (a) temperature and (b) lamellar thickness (*l*). In (c), the schematic shows the differences between the extended chain conformation in cyclic polymers and the once-folded chain conformation in linear polymers. Figure 9 is adapted from ref 26. Copyright 2018 American Chemical Society.

Zaldua et al.^26^ determined the *T*_*m*_° using the GT^153^ and modified GT equations for cyclic polymers.^159^ SAXS experiments allowed the determination of *l* as *l* = *X*_*v*_·d*. The resulting *T*_*m*_ vs 1/*l* plots revealed *T*_*m*_° (linear) ≈ 159 °C and *T*_*m*_° (cyclic) ≈ 164 °C, confirming that cyclic samples reach higher *T*_*m*_°. Figure 9b shows the SSA traces plotted versus *l*, while Figure 9c schematically depicts the extended-chain (cyclic) and once-folded (linear) conformations. Fraction 1 for cyclic samples approaches *l* ≈ 20–30 nm, close to the theoretical limit for a fully extended cyclic chain (∼20.5 nm).

Overall, these results demonstrate that topology, via its impact on entanglement density and diffusional mobility, controls the annealing capacity revealed by SSA: cyclic polymers generate thicker lamellae and higher-*T*_*m*_ fractions than linear analogues despite the latter’s thermodynamic potential for longer extension.

#### 3.2.2 Topology Effects: Threading Effect in Cyclic/Linear Blends

Cyclic polymers often contain trace linear contaminants that can dramatically alter their crystallization kinetics. Blending cyclic polymers with small amounts of linear chains generates the so-called threading effect.^23,160,161^ Linear chains can reptate through cyclic ones, increasing effective entanglement density and hindering diffusion or lamellar growth.^162–165^

López et al.^23^ examined *c*-PCL/*l*-PCL blends (95/5, 90/10, 80/20 wt %) at two molecular weights (*M*_*n*_ = 3 and 12 kg/mol). The cyclic samples were synthesized by click chemistry.^166,167^ These blends do not follow simple mixing rules: even 5–10 wt % of *l*-PCL significantly depresses *T*_*c*_ and *T*_*m*_, reduces crystallinity and spherulitic growth, and slows overall crystallization.

The SSA behavior is shown in Figure 10b, where neat *c*-PCL exhibits a high annealing capacity, fractionating up to Fraction 1, while *l*-PCL fractionates only up to Fraction 3. The addition of merely 10 wt % of linear chains suppresses the highest fraction due to the threading effect. Figure 10a illustrates this phenomenon: linear chains thread through cyclic rings, creating additional entanglement points that limit mobility and diffusion. As a result, the annealing capacity of *c*-PCL in the blend is drastically reduced.

**Figure 10:**
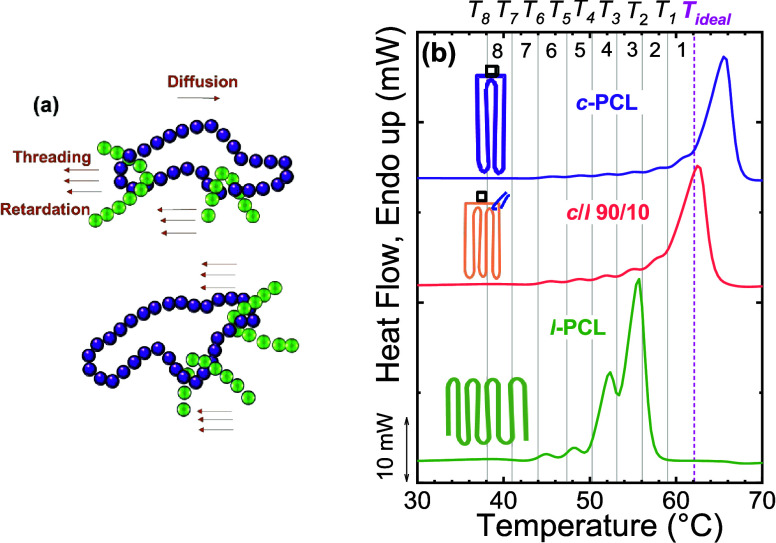
(a) Schematic representation of the threading effect; (b) SSA profile for *l*- and *c*-PCL (of 3 kg/mol) and the 90/10 *c*/*l*-PCL blend. The Δ*T*_*s*_ = 5 °C. Figure 10 is adapted with permission from ref 23. Copyright 2016 the Royal Society of Chemistry.

SSA experiments, therefore, provide a sensitive means to detect even minor contamination or topological coupling in cyclic/linear systems, quantifying how the threading effect modifies chain diffusion and crystal perfection.

#### 3.2.3 Topology Effects: Linear, Star, and Comb Copolymers

Beyond cyclic architectures, branching introduces additional topological restrictions that strongly affect SSA fractionation. Pérez-Camargo et al.^128^ studied poly­(ethylene sulfide)-*co*-(propylene sulfide) (PS*_*x*_*-ES*_*y*_*) copolymers with varying topologies: linear (*L*), star (*S*), and comb (*C*), in which only the ES block crystallizes.

Figure 11a and Figure 11b show the nonisothermal cooling and heating DSC scans for linear, with different molecular weights, quantify through the degree of polymerization (DP), and star copolymers (broad, overlapping peaks) with various molecular weights and number of arms. In Figure 11c, the main thermal transitions and their enthalpies (Figure 11d) are plotted as a function of the number of arms for all the cases, including the comb copolymers, evidencing how the number of arms affects differently depending on the specific topology (star vs combs).

**Figure 11:**
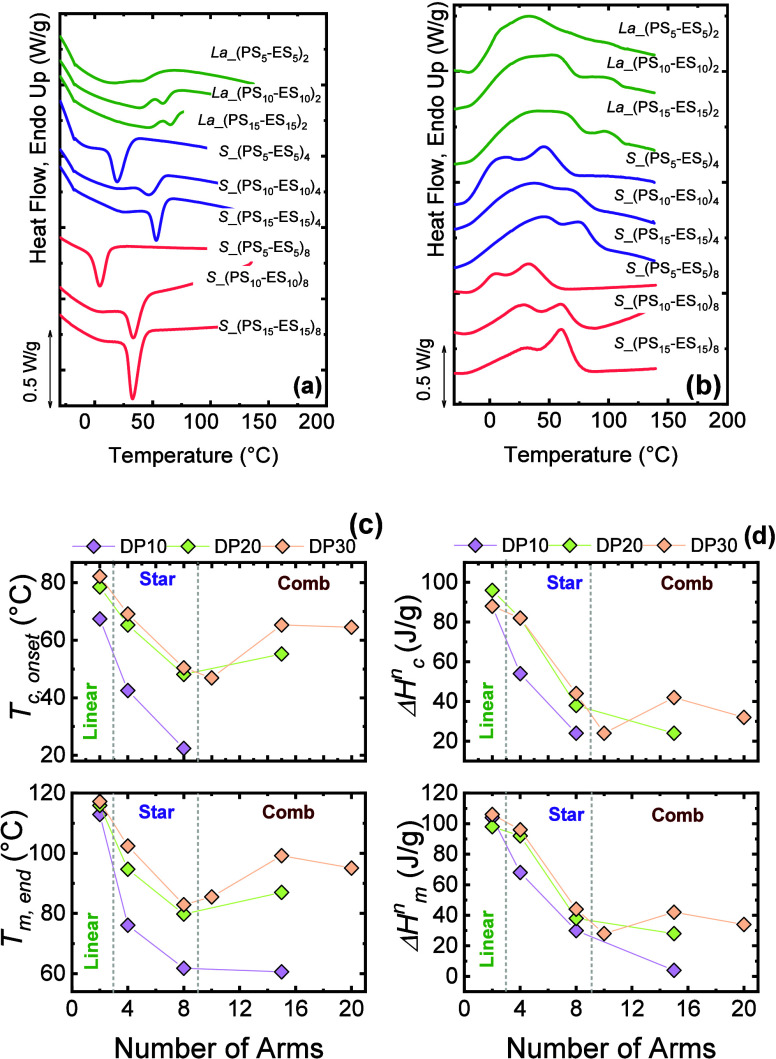
(a) Cooling and (b) second heating DSC scans for linear (*L*_*a*_) and Star (*S*) copolymers. (c, d) Crystallization onset temperatures (*T*_*c,onset*_) and end melting temperatures (*T*_*m,end*_), (d) crystallization (Δ*H* ^*n*^_*c*_) and melt (Δ*H* ^*n*^_*m*_) normalized enthalpies as a function of number of arms, for linear, star, and comb samples with different DPs. The solid lines represent guides to the eye. The dashed lines separate the behavior of the linear, star, and comb samples. Figure 11 is adapted from ref 128. Copyright 2019 American Chemical Society.

Applying SSA (Figure 12) yields thermally fractionated sharper melting peaks that resolve the topological effects. Linear copolymers (Figure 12a, top), free of topological constraints, consistently exhibit Fraction 1 (the highest fraction), and its area increases with DP. Star copolymers (Figure 12a, bottom) crystallize only on the arms (the backbone is atactic). Convergent crowding at the branching points constrains conformations, reduces flexibility and diffusion, and suppresses Fraction 1; as the number of arms increases, the maximum fraction shifts to 2 and 4 (4- and 8-arm stars, respectively).

**Figure 12:**
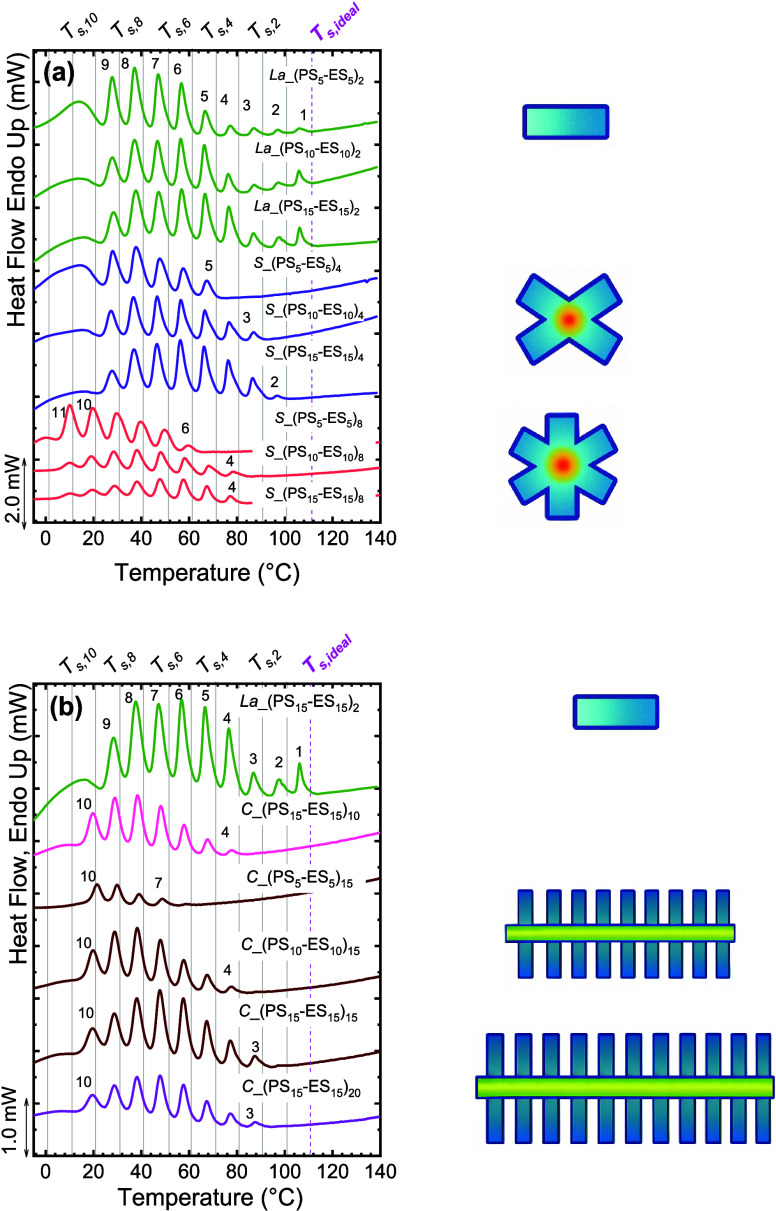
SSA profiles of (a) *L* and *S* copolymers and (b) *L* and *C* copolymers. The vertical lines represented the employed *T*_*s*_. On the right-hand side of the panels, the different topologies are represented as cartoons. Figure 12 is adapted from ref 128. Copyright 2019 American Chemical Society.

Comb copolymers (Figure 12b) experience parallel crowding, permitting partial intramolecular ordering; at high DP (e.g., DP = 30), the highest fraction re-emerges and even increases with arm number-opposite to stars.

Thus, SSA demonstrated that branching topology, via geometrical crowding and chain confinement, dictates the distribution and perfection of crystalline lamellae.

#### 3.2.4 Topology Effects: Star Architectures and Interdigitation Phenomena

The effect of star topology on SSA fractionation has been revisited using P3HB homopolymers.^44^ Gace et al.^44^ synthesized isotactic (*it-*), syndio-rich (*sr-*), and iso-rich (*ir-*), and four-arm star-shaped (*s4*) P3HBs and linear (*l*) analogues of similar molar mass via stereochemically controlled ROP of cyclic diolides with yttrium salen complexes catalysts, providing a new perspective on how architectural control modulates thermal fractionation and crystalline assembly.

SSA experiments revealed that both linear and star isotactic P3HBs exhibit limited fractionation; however, the star polymer shows a distinct high-temperature melting fraction (≈ 174 °C) absent in the linear analogue (Figure 13a). This additional endotherm is attributed to interdigitation between arms of neighboring stars, which locally enhances lamellar thickness despite lower global crystallinity. The amorphous star core is excluded from the lattice, while extended arms interpenetrate adjacent lamellae, producing a percolated crystalline network, because stars tend to have their arms in a more stretched-out conformation. Interestingly, this is a different effect from that observed with the star-polysulfides, in which the annealing capacity was reduced relative to the linear analogs (Figure 12a).

**Figure 13:**
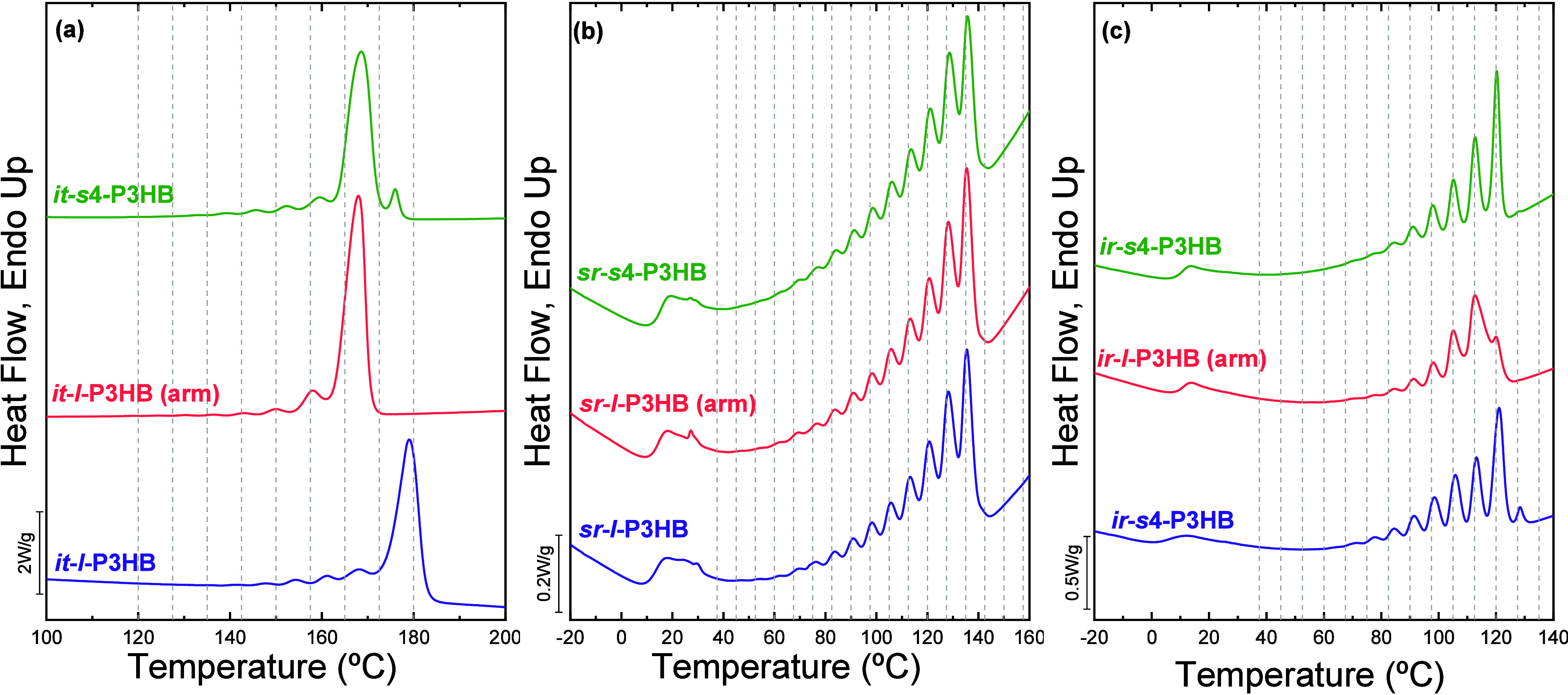
Final DSC heating scans after SSA for (a) isotactic linear vs four-arm star P3HBs, highlighting the ≈174 °C high-*T*_*m*_ fraction exclusive to stars; (b) for *sr*-rich P3HB stars vs linear analogues; and (c) for *ir*-rich P3HB stars vs linear analogs. Figure 13 is Adapted from ref 44. Available under CC-BY 3.0 license. Copyright 2025 the Royal Society of Chemistry.

In *ir*- and *sr*- star P3HBs, a greater number of well-defined fractions is observed (Figure 13b), consistent with enhanced segregation driven by stereodefects; still, total crystallinity remains lower than in linear counterparts due to restricted mobility at the star core.

In summary, SSA exposes two complementary consequences of star topology: (i) reduced overall crystallinity by constrained diffusion, and (ii) emergence of high-*T*_*m*_ fractions caused by local arm interdigitation, i.e., enhanced local lamellar thickening.

### 3.3 Nanocomposites: Supernucleation, Pre-freezing, and Antinucleation Effects

The incorporation of nanoscale fillers into semicrystalline polymers profoundly alters their crystallization behavior, not only by accelerating nucleation but also by modifying the crystal stability and fractionation pattern revealed by SSA. Depending on the nature of polymer–filler interactions, nanofillers may act as supernucleating agents, generating crystals of exceptional stability, or as antinucleating surfaces, hindering nucleation and lamellar thickening. In other cases, strong polymer–substrate interactions can produce interfacial crystalline layers that melt above the polymer’s equilibrium melting temperature, a phenomenon known as prefreezing.

These distinct behaviors are uniquely discernible by SSA technique, which allows the detection of both annealable and nonannealable crystalline entities, thus providing a powerful framework to rationalize how confinement, adsorption, and interfacial energy determine crystallization in polymer nanocomposites.

#### 3.3.1 Supernucleation and Pre-freezing Effect: Fractionated vs Unfractionated Thermal Transitions

The interaction between polymer chains and nanoscale surfaces can promote distinct interfacial crystalline organizations that SSA can disentangle with exceptional clarity. Depending on the interfacial energy landscape, nanofillers may generate fractionable extended-chain crystals or unfractionable interfacial layers whose melting exceeds the polymer’s *T*_*m*_°.

A first paradigmatic case was reported by Colonna et al.,^126^ who prepared poly­(butylene terephthalate) nanocomposites with reduced graphene oxide without annealing and annealed at 1700 °C (pCBT + 10 wt % RGO and pCBT + 10 wt % RGO_1700_). The RGO acted as a supernucleating agent, producing nucleation efficiencies (*NE*) up to 270%. SSA experiments (Figure 14 for pCBT + 10 wt % RGO_1700_) revealed a high-temperature endotherm (∼250 °C) approaching *T*_*m*_° that could still be fractionated when scanned between 252 and 197 °C. Hence, these crystals correspond to real lamellae capable of thickening, not to adsorbed layers. WAXS confirmed persistent α-form reflections above the normal melting range, evidencing extended-chain lamellae (20–32 nm) nucleated at the polymer/RGO interface four to six times thicker than those of neat pCBT. Thus, this system exemplifies true supernucleation leading to fractionable, highly stable lamellae.

**Figure 14:**
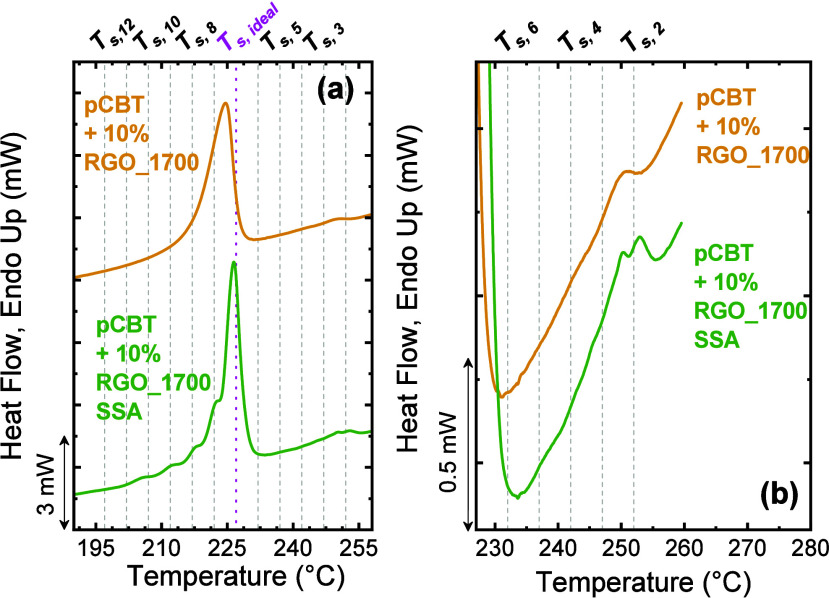
(a) SSA profile vs standard second heating scan for the pCBT + 10%RGO_1700_. In (b), the fractionated region at high temperatures is zoomed in, showing the formation of fractions. Figure 14 is adapted from ref 126. Copyright 2017 American Chemical Society.

A second complementary study, the PE-*g*-SiO_2_ nanocomposites, prepared through a “grafting to” method with varying grafting densities and molecular weights, bridges the gap between classical supernucleation and interfacial confinement.^102^ Depending on the grafting density, *T*_*c*_ can fall below that of neat PE, or alternatively, a fractionated crystallization emerges, displaying three components: low-, middle-, and high-crystallization peaks (LCP, MCP, and HCP, respectively).

In SSA experiments, at low molecular weight or low grafting density, covalent anchoring imposes both spatial and chain confinement, leading solely to the formation of the highly confined LCP component. By contrast, at higher molecular weight and grafting density, the confinement effect weakens, allowing some grafted chains to form the MCP and HCP populations that crystallize more readily. Meanwhile, the overall nucleation effect strengthens with increasing grafting density, producing additional high-temperature melting peaks in SSA associated with lamellae forming close to the SiO_2_ surface. The grafting simultaneously restricts chain mobility and enhances heterogeneous nucleation, thus generating confined yet extended lamellae that melt above the bulk *T*_*m*_.

Unlike the unfractionable prefrozen layers discussed below, these high-*T*_*m*_ populations remain fractionable by SSA, confirming that they correspond to genuine crystals stabilized by interfacial regularity rather than adsorption. This case elegantly demonstrates how confinement and grafting can mimic supernucleation without leading to prefreezing. It is important to note, however, that not every supernucleation event produces a high-temperature peak, underscoring that the outcome depends sensitively on the interplay among grafting density, molecular weight, and interfacial energy.

Finally, Fina et al.,^30^ and more recently Zhao et al.^41^ extended the concept of the interplay between supernucleation and interfacial confinement to PCL/graphene-based nanopaper, unveiling a hierarchical organization of crystalline populations (Figure 15A–D) governed by polymer–graphene interactions. Nonisothermal DSC traces revealed a large increase in *T*_*c*_ (≈47 °C vs 28 °C for neat PCL, Figure 15a), demonstrating the strong nucleating power of GNPs and rGO. The four endothermic peaks (Figure 15b) at ≈57 °C (A), 75 °C (B), 85 °C (C), and 120 °C (D) reflect progressively stronger interfacial constraints.

**Figure 15:**
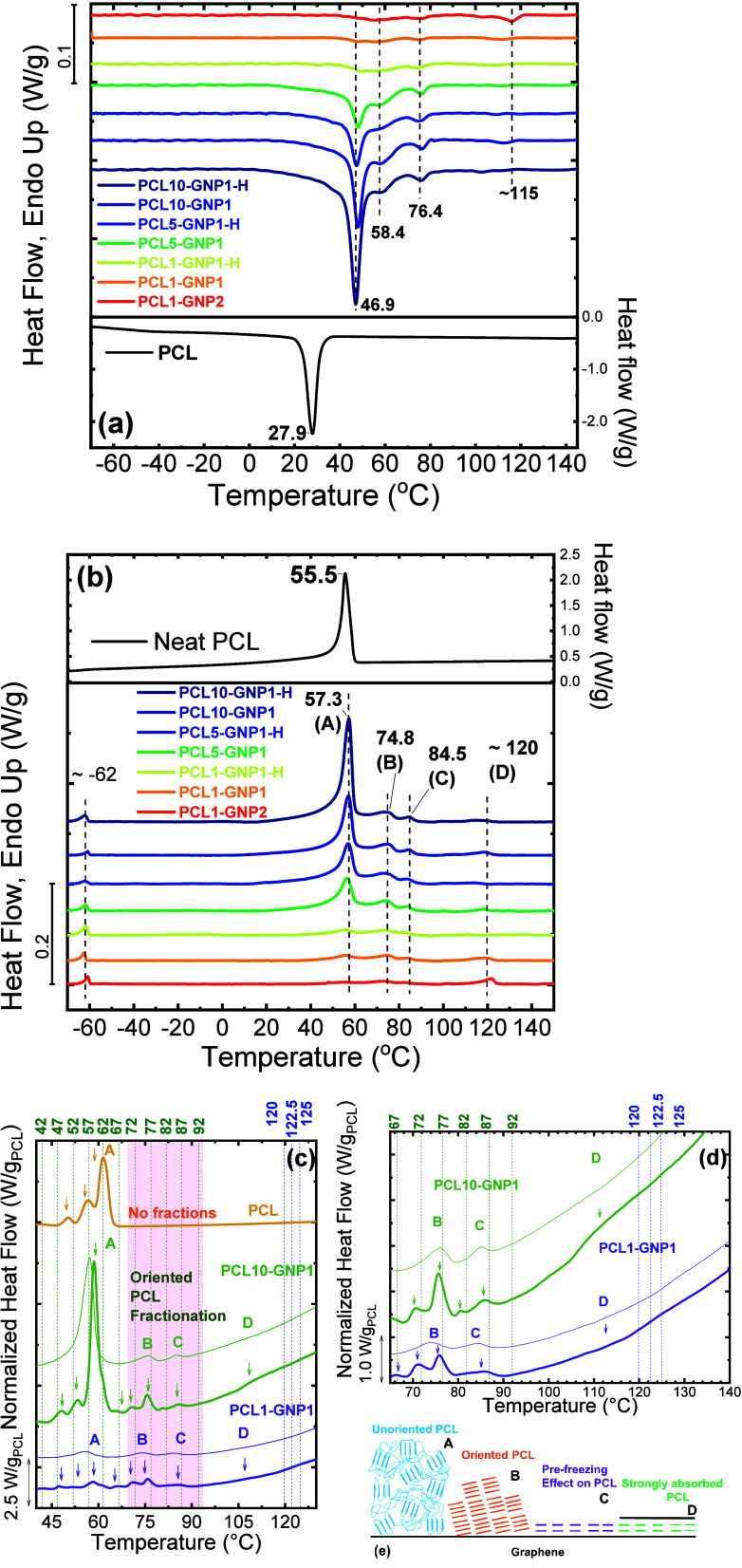
(a) Cooling and (b) second heating DSC scans. In (b), the endothermic peaks are identified as A, B, C and D. (c) SSA profile of PCL, PCL10-GNP1, and PCL1-GNP1. The fractionation at high temperatures and with Δ*T*_*s*_ = 2.5 °C is indicated with blue lines, whereas the second fractionation at lower temperatures and Δ*T*_*s*_ = 5 °C is shown with green lines. The vertical lines in (c) and (d) indicate the *T*_*s*_ employed (see the top of the panels). The peaks have been labeled A, B, C, and D according to their nature. (d) Zoom of the SSA profile for Peaks B–D. In both (c) and (d), the thinner lines indicate the DSC traces of unfractionated samples. (e) Illustration of the possible origin of Peaks A–D. Note that the weight of the PCL normalized all the curves of each sample. Figure 15 is adapted from ref 30. Copyright 2021 American Chemical Society.

SSA fractionation (Figure 15c,d) was conducted to disentangle the origins of the multiple melting peaks and identify which crystalline populations could thicken upon controlled annealing. To this end, a combined SSA protocol was applied: the first series of steps employed a fine temperature interval (Δ*T*_*s*_ = 2.5 °C) at high *T*_*s*_ to resolve the narrow high-temperature transitions (Peaks D-C), while a second series used a broader interval (Δ*T*_*s*_ = 5 °C) at lower *T*_*s*_ to capture the standard PCL fractionation behavior. This design minimized possible overlap or degradation and allowed a direct correlation between thermal and structural responses across the four crystallization populations. The resulting SSA profiles revealed distinctive behaviors (Figure 15c,d).

SSA fractionation (Figure 15c,d) confirmed that:

*Peaks A and B* are fractionable: *Peak A* corresponds to bulk-like unoriented crystals, while *Peak B* corresponds to oriented lamellae aligned parallel to the graphene surface. This was corroborated by WAXS and GIWAXS, where new reflections, i.e., (102)/(003) and (103), and an anisotropic azimuthal distribution of the (110) reflection appeared, evidencing epitaxial or surface-induced orientation. Details on the 2D WAXS patterns acquired in transmission and GIWAXS geometries are provided in ref 41.

*Peaks C and D* are unfractionable: *Peak C* originates from a prefreezing phenomenon, i.e., a crystalline prewetting layer thermodynamically stable above *T*_*m*_ *°. Peak D* corresponds to strongly adsorbed or intercalated PCL layers confined between graphene sheets that cannot reorganize during SSA. Zhao et al.^41^ further demonstrated that the stability of these prefrozen layers increases with molecular weight, consistent with the thermodynamic model of Thurn-Albrecht et al.,^168^ where the free-energy balance between surface and bulk phases defines the maximum stability temperature of the prefrozen layer.

Altogether, the combined evidence from SSA and WAXS establishes a continuum of interfacial crystallization regimes: (a) Fractionable extended lamellae (supernucleation), (b) Fractionable oriented lamellae (surface-induced order), (c) Unfractionable prefrozen layers, and (d) Unfractionable adsorbed/intercalated layers. This hierarchy shows how SSA and structural probes jointly uncover the full spectrum of polymer-nanofiller interactions, from enhanced nucleation to molecular immobilization. Importantly, such insight transcends the conventional view of nanocomposites as mere nucleation enhancers, revealing that interfacial thermodynamics can drive polymers into metastable, above-equilibrium crystalline states.

#### 3.3.2 Antinucleation Effect: Specific Interactions

While many nanofillers accelerate crystallization through surface-induced nucleation, strong or specific polymer–filler interactions can instead suppress nucleation, limiting lamellar thickening and producing what can be described as an antinucleation effect. SSA proves particularly powerful at distinguishing these situations because it sensitively reveals the depletion of higher-melting fractions that would otherwise result from successful crystal thickening and self-nucleation.

A clear example was reported by Pérez-Camargo et al.,^22^ who synthesized lignin-grafted PCLs (lignin-*g*-PCL) containing different lignin contents (2–37 wt %) and average arm lengths (AALs). For clarity, the samples were denoted as PCL*_*x*_^y^* where *x* indicates lignin content and *y* corresponds to the AAL of the grafted PCL chains. At low lignin levels (<18 wt %), lignin behaved as an excellent or even supernucleating agent (Figure 16a), yielding *NE*s^169^ near or above 100%, but without showing high-temperature peaks as in the cases shown above.^30,41,102,126^ Accordingly, both *T*_*c*_ and *T*_*m*_ increased, and crystallization accelerated. However, beyond ≈ 18 wt % lignin, the trend reversed dramatically: *T*_*c*_ and *T*_*m*_ (Figure 16b) decreased, crystallinity dropped, and overall crystallization kinetics slowed, revealing a transition from supernucleation to antinucleation. These trends are plotted as a function of lignin content in Figure 16.

**Figure 16:**
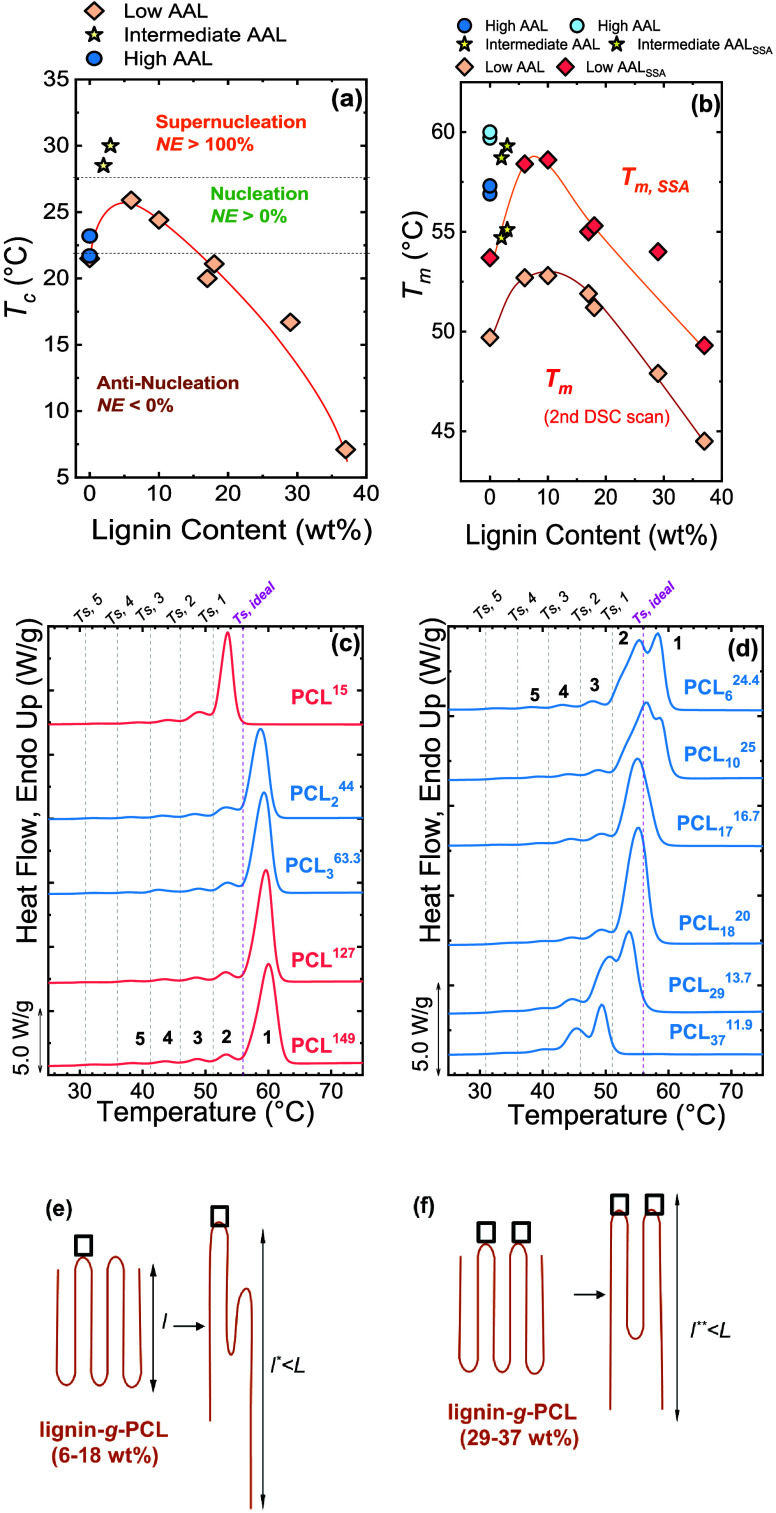
(a) *T*_*c*_ variation as a function of lignin content; (b) comparison of *T*_*m*_ obtained after SSA (*T*_*m,SSA*_) and before SSA (*T*_*m*_) as a function of lignin content. In (a), the antinucleation, nucleation, and supernucleation effect regions are indicated. In (b), a maximum in both *T*_*m*_ and *T*_*m,SSA*_ is detected in line with (a). (c) SSA profile for neat PCLs and PCL_2_^44^, PCL_3_^63.3^ (low lignin content), and (d) SSA profile for lignin-*g*-PCL with lignin contents >3 wt %. The vertical lines in (c) and (d) represent the *T*_*s*_ employed, and the generated fractions are labeled. The neat PCLs are indicated in red and the lignin-*g*-PCL materials in blue. In (e) and (f), a possible way for PCL chains in lignin-*g*-PCL to undergo thickening during annealing is schematically illustrated. The *not*-*to*-*scale* square represents hydrogen bonding between PCL and lignin. Acting like physical cross-links, they prevent chain segments around them from entering PCL crystals: (e) intermediate lignin contents with a low density of hydrogen bonds and (f) high lignin contents with a higher density of hydrogen bonds. Figure 16 is adapted with permission from ref 22. Copyright 2015 John Wiley and Sons.

SSA heating scans elegantly captured this evolution, as shown in Figure 16c and Figure 16d. All materials were fractionated using the same *T*_*s,ideal*_ = 56 °C, Δ*T*_*s*_ = 5 °C, *t*_*s*_ = 5 min, and 6 steps (yielding 5 fractions).

For neat PCLs and lignin-*g*-PCLs with low lignin content (PCL_2_^44^ PCL_3_^63.3^), the SSA profiles resembled those of high-molar-mass PCLs, with clear multiple fractions indicating efficient lamellar thickening. At higher lignin contents (6–37 wt %), however, a progressive depletion of the highest-melting fraction was observed, until it vanished near 17–18 wt %. At 29 wt %, fraction 2 dominated the SSA profile, and at 37 wt %, even that fraction disappeared, leaving only fraction 3 (Figure 16d). Figure 16b also plots *T*_*m,SSA*_ (and *T*_*m*_) versus lignin content (Figure 16b), revealing a steady decline consistent with the *T*_*c*_ (Figure 16a) and kinetic data.^22^ The antinucleation behavior was attributed to intermolecular hydrogen bonding between PCL carbonyl groups and lignin’s phenolic and aliphatic hydroxyls, as previously reported by Laurichesse and Averous.^170^ These hydrogen bonds act as physical cross-links, hindering chain diffusion and crystal thickening. Rheological experiments confirmed the presence of cross-link-like interactions that persist even after successive annealing cycles.

SSA cartoons (Figure 16e,f) depict how, at intermediate lignin contents, isolated H-bond sites slightly restrict chain mobility, whereas at higher contents the dense network of interactions effectively immobilizes chain segments, preventing lamellar growth. This molecular locking mechanism parallels the threading limitation observed in cyclic/linear PCL blends^23^ and other branched topologies,^128^ where topological constraints rather than chemical cross-links restrict lamellar reorganization.

Thus, in lignin-*g*-PCL systems, the interplay between heterogeneous nucleation and intermolecular bonding governs the SSA outcome. At low lignin content, nucleation dominates, yielding near-ideal efficiency; at high content, H-bond formation prevails, generating an antinucleating environment. SSA directly visualizes this competition by translating microscopic intermolecular interactions into macroscopic thermal fractionation patterns. Such insight underlines the conceptual strength of SSA in identifying hidden interfacial or molecular constraints that suppress crystallization despite the presence of potential nucleating sites.

### 3.4 SSA as a Tool to Enhance the DSC Signal

The powerful crystal-thickening ability of the SSA protocol can be exploited not only to map lamellar distributions but also to magnify weak transitions that remain hidden under conventional DSC conditions. Two paradigmatic cases are solid–solid transitions in aliphatic polycarbonates and the subtle crystallization of segmented polyurethanes.

#### 3.4.1 Solid–Solid Transitions

Solid–solid transitions provide a fascinating playground for testing the sensitivity of SSA toward conformational rearrangements within crystals. Pérez-Camargo et al.^33,171^ reported δ ↔ α transitions in poly­(hexamethylene carbonate) (PC6) and poly­(octamethylene carbonate) (PC8), which are almost invisible in standard DSC scans. By combining isothermal + nonisothermal protocols^171^ and, later, SSA fractionation,^33^ the authors amplified the endothermic δ → α signal and clarified its nature as a reversible order–disorder conversion between more efficiently packed δ-chains and less ordered α-chains.

Figure 17a and Figure 17c compare the second-heating and SSA profiles for PC6 (Figure 17a) and PC8 (Figure 17c). Whereas conventional scans show broad or barely discernible events, SSA fractionation (*T*_*s,ideal*_ = 56 °C for PC6 and 58 °C for PC8; Δ*T*_*s*_ = 5 °C) generates discrete, intense peaks around 20 °C (PC6) and 35 °C (PC8), corresponding to the δ → α transition. These enhanced, unfractionated peaks, two- to 3-fold larger in enthalpy than the conventional ones, emerge because annealing produces highly stabilized lamellae that transform cooperatively. WAXS confirmed the structural origin of the signal, while FT-IR correlated it to the methylene conformational shift from trans–gauche disorder (α) to ordered all-trans (δ).

**Figure 17:**
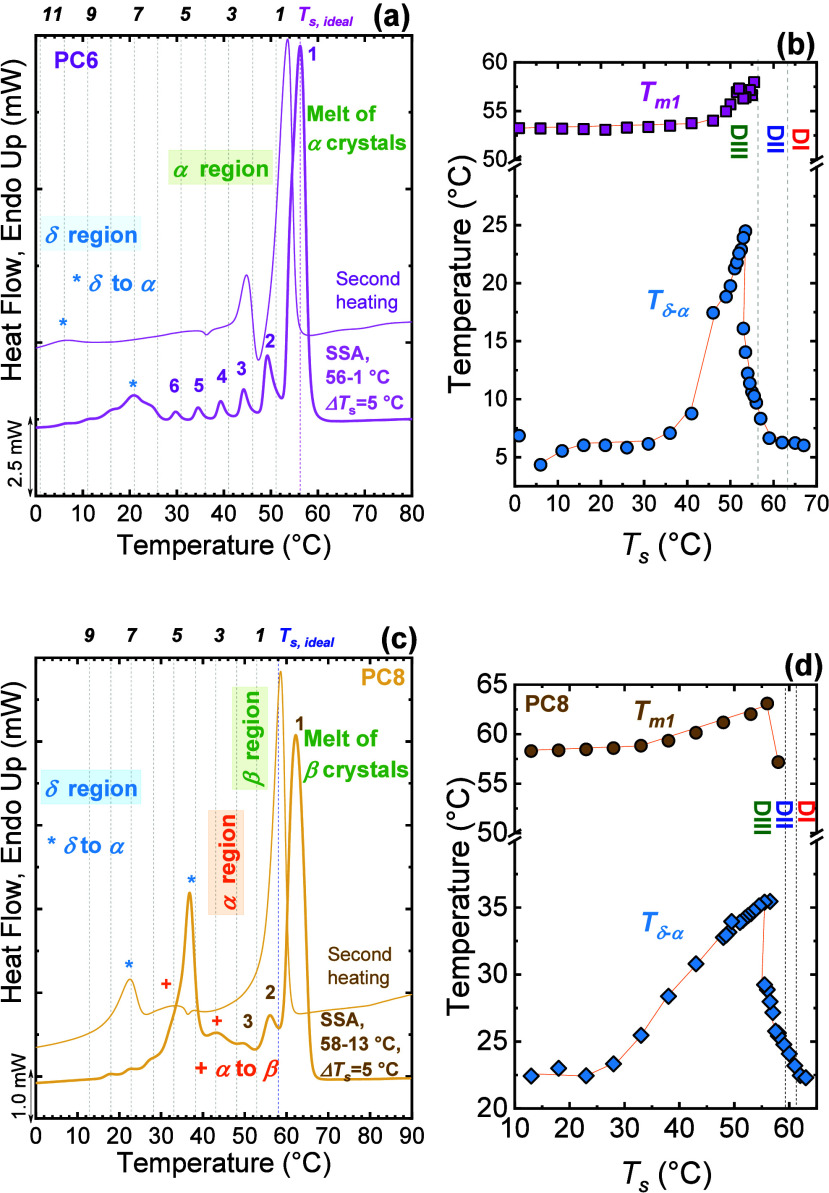
Comparison of the second heating DSC scan and SSA profile for (a) PC6 and (c) PC8. The vertical lines in (a) and (b) indicate the used *T*_*s*_, and the generated fractions and the position of the δ to α transition is labeled. (b) and (d) show the evolution of the endothermic δ to α transition, *T*_δ‑α_, and the highest melting point, *T*_*m*1_, as a function of *T*_*s*_, for (b) PC6 and (d) PC8. The *T*_δ‑α_ and *T*_*m*1_ values were obtained from SN experiments at selected *T*_*s*_, including those used in SSA experiments. The vertical dashed line separates *Domains I, II,* and *III*. For clarity, the plotted *T*_*m*1_ data corresponds to selected *T*_*s*_ values. Figure 17 is adapted from ref 33. Copyright 2021 American Chemical Society.

Varying the SSA conditions (starting *T*_*s*_, Δ*T*_*s*_, number of steps) demonstrated that the transition temperature follows the crystal stability of the thickest lamellae. This relationship becomes particularly evident in SN experiments, where plotting the solid–solid transition temperature (*T*_δ‑α_) and the highest *T*_*m*_ (*T*_*m1*_) as a function of *T*_*s*_ (Figure 17b,d, including those *T*_*s*_ used in SSA experiments) shows both quantities following the same trend: the more perfect and thicker the lamella, the higher the *T*_δ‑α_. Such coupling between lamellar perfection and transition temperature contrasts sharply with the behavior of Brill-type transitions observed in polyamides, where conformational and lamellar order remain largely decoupled. Hence, the SSA protocol acts as a precision amplifier of subtle solid–solid transformations, sensitively linking molecular conformational order to lamellar stability and offering a unique thermodynamic fingerprint of crystalline perfection.

#### 3.4.2 Thermoplastic Polyurethanes (TPU): Fractionation of Hard-Block Distributions

Conventional DSC scans of thermoplastic polyurethanes (TPUs) typically display very broad and poorly resolved melting endotherms, arising from the wide distribution of hard-segment lengths and the coexistence of amorphous and semicrystalline microdomains. This feature severely limits any detailed interpretation of their crystallization or phase-segregation behavior.

The SSA protocol overcomes this limitation by promoting controlled lamellar reorganization. Fernández-d’Arlas et al.,^129^ first demonstrated that TPUs (Figure 18), with different combinations of methylene diphenyl diisocyanate (MDI) and 1,4-butanediol (BD) as hard phase, and polyols, either adipic polyester or polytetrahydrofuran (polyether), as soft segments (namely PUeth30, PUest33, PUeth43) can be successfully fractionated without degradation by applying an SSA cycle with *T*_*s,ideal*_ = 210 °C, Δ*T*_*s*_ = 10 °C, and a shortened annealing time *t*_*s*_ = 1 min at each step. This short time, validated through preliminary SN tests, prevents thermal degradation above 200 °C while still yielding excellent resolution. The resulting series of discrete, intense melting fractions (Figure 18b) revealed distinct populations of hard-segment crystals (MDI-BD) embedded in different microdomains, which contrast sharply with the single broad endotherm observed in the conventional nonisothermal second-heating scans (Figure 18a). Similar comparisons have been reported and plotted (Figure 18c) in the literature.^133,135,136^

**Figure 18:**
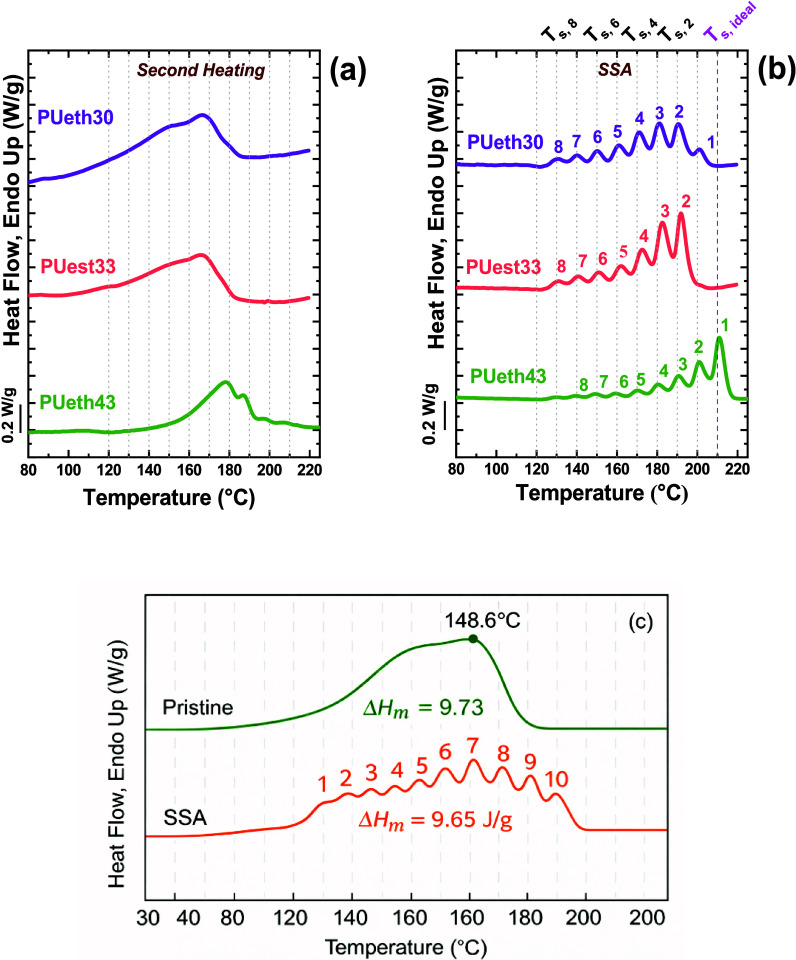
Comparison of (a) second heating DSC scans and (b) SSA profiles for various TPUs or PUs. The vertical lines indicate the different *T*_*s*_ used (in (a), they are included for comparison purposes), and the fractions are labeled with numbers. (c) directly compares the second heating and the SSA profile for a TPU with a low hard-segment content. (a) and (b) are adapted with permission from ref 129. Copyright 2020 John Wiley and Sons. (c) is adapted with permission from ref 136. Copyright 2023 Springer Nature.

Compared with the featureless second-heating scans (Figure 18a), the SSA-resolved profiles (Figure 18b) display well-defined peaks separated by ≈ 10 °C, which can be directly related to a hierarchy of lamellar thicknesses. This improved thermal resolution translated into clear morphological refinement: AFM topography images showed much thicker and sharper lamellae in the fractionated samples (Figure 19a,b), while WAXS and SAXS patterns exhibited better-defined MDI-BD crystalline reflections and an increase in long-period order (Figure 19c,d).

**Figure 19:**
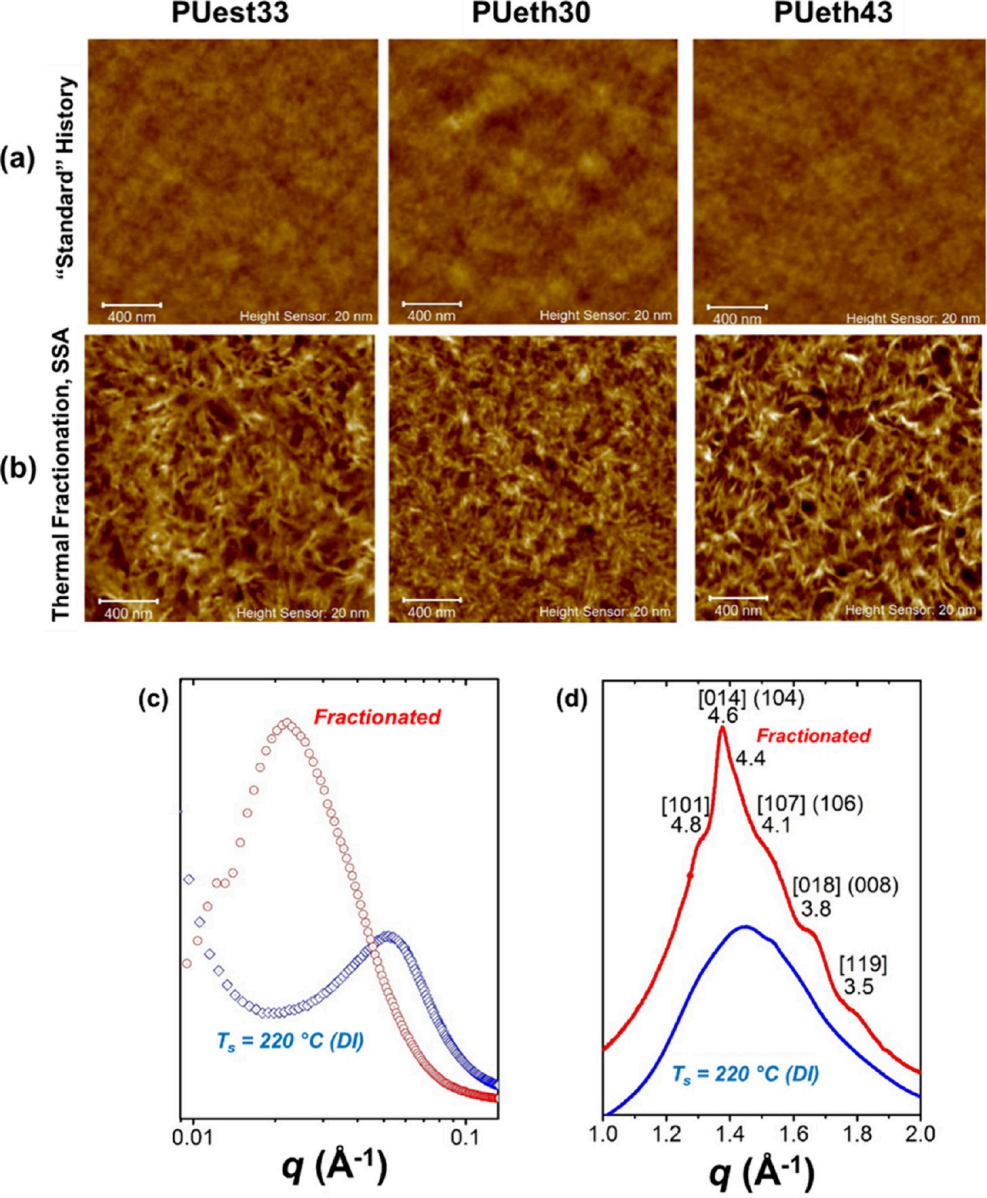
Comparison of unfractionated and fractionated samples through AFM topography images of (a) unfractionated samples and (b) fractionated samples (SSA protocol was applied without the final heating); (c) WAXS and (d) SAXS of PUeth30 before and after fractionation. Figure 19 is adapted with permission from ref 129. Copyright 2020 John Wiley and Sons.

Building upon this approach, Wang et al.,^136^ Gao et al.,^133^ and Liu et al.^135^ systematically expanded the application of SSA to TPUs by varying the hard-segment content (HSC), the reaction temperature, and the processing method (hand vs machine casting), respectively. All these studies employed short SSA cycles (*t*_*s*_ = 1 min, Δ*T*_*s*_ = 10 °C) to prevent degradation while achieving outstanding resolution. Together, they demonstrated that increasing the HSC, lowering the synthesis temperature, or switching from batch to continuous production produces measurable changes in hard-block topology and lamellar hierarchy, differences that remain invisible in standard DSC scans but become evident through SSA fractionation as discrete, 10 °C-spaced melting peaks (Figure 18c).

For instance, TPUs with lower reaction temperatures^133^ or higher HSC^136^ contained a minor population of longer MDI-BD blocks that crystallized earlier and promoted superstructures scattering visible light, thereby reducing transparency. In contrast, machine-cast TPUs^135^ with narrower hard-block distributions exhibited thicker and more uniform lamellae (*l*_*c*_ ≈ 14 nm vs 12 nm for hand-cast samples) and superior elasticity and recovery, as confirmed by SSA-resolved melting profiles. The combined use of SSA, AFM, WAXS, and SAXS revealed that thermal fractionation not only increases the overall crystallinity (up to ≈7%) but also refines the domain periodicity and enhances the detectability of crystalline reflections.^129,136^

In conceptual terms, TPUs epitomize the “polymer-by-process” paradigm:^135^ the delicate interplay between synthesis and postprocessing conditions governs chain topology, morphology, and macroscopic performance. SSA thus acts as a signal-amplifying bridge, transforming broad, ambiguous DSC endotherms into quantifiable, structure-specific melting sequences that can be directly correlated with morphological and mechanical properties such as modulus, elasticity, and transparency.^129,133,135,136^

### 3.5 SSA for Determining the Composition and Structural Evolution of Recycled Polymeric Materials

The application of SSA to recycled polymers has evolved from a purely academic thermal-fractionation technique into a practical analytical tool for the circular-economy era. Recycled materials often contain multiple semicrystalline components, such as PP, HDPE, and LDPE, with overlapping melting domains that hinder precise compositional analysis. Traditional methods like TREF, FTIR, or ^13^C NMR provide accurate results but are slow and instrumentally demanding.

By contrast, SSA offers a thermal-fingerprint approach that can be performed directly on a DSC, enabling both quantitative determination of blend composition and monitoring of lamellar reorganization during mechanical recycling. Unlike conventional DSC melting or crystallization experiments, which often collapse the thermal response of complex recycled blends into one or two broad and overlapping endotherms, SSA introduces a controlled thermal history that progressively amplifies lamellar stability differences. Through successive self-nucleation and annealing steps, crystalline populations that remain unresolved under standard DSC conditions become thermally separated, transforming a single melting peak into a diagnostic fractionation fingerprint.

This section outlines the progressive adaptation of SSA, from the original coupled SN + SSA protocols for mixed polyolefins, to accelerated single-fraction schemes and calibration-based quantification of commercial recyclates, and finally to its use in tracking structural evolution in biobased systems, underscoring its versatility as a bridge between structure, processing history, and recyclability.

#### 3.5.1 From Coupled SN-SSA Protocols to Single-Fraction Fast Screening

The pioneering work by Carmeli et al.^5^ represents the first systematic use of thermal fractionation to analyze recycled PE/PP blends. Their study addressed a common challenge in polyolefin recycling: mutual contamination between PP and PE phases, which strongly affects melt behavior, crystallinity, and final mechanical performance.

To reproduce realistic situations while maintaining controlled compositions, the authors prepared two types of materials: (i) model blends of PP homopolymer (homo) or heterophasic PP (het, containing rubber inclusions) with LDPE (ρ = 0.923 g/cm^3^) and HDPE (ρ = 0.945 g/cm^3^) in overall 60/40 and 40/60 PP/PE ratios, where the PE phase itself consisted of equal proportions of LDPE and HDPE; and (ii) commercial recycled blends from distinct sources, postconsumer (PCR1, PCR2) and postindustrial (PIR1, PIR2). According to supplier FTIR data, PCR1 and PIR1 contained roughly 40/60 and 10/90 PE/PP, respectively, while PCR2 and PIR2 had unknown compositions.

To characterize all systems, Carmeli et al.^5^ developed a coupled SN and SSA (SN + SSA) protocol, schematically illustrated in Scheme 5a and Scheme 5b.

**Scheme 5:**
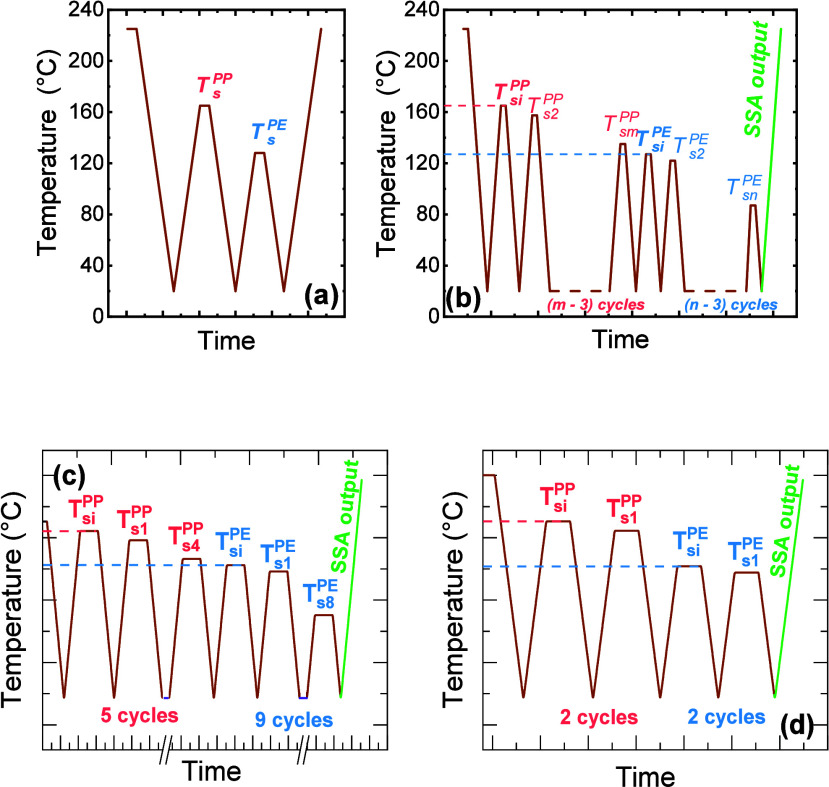
Coupled (a) SN and (b) SSA Protocol designed for (a) Self-nucleated and (b) Fractionated Blends of PE and PP; (c) Fractionation Program (Multi-fraction Protocol) implemented and designed by Carmeli et al.,^5^ which uses 14 cycles to obtain 8 fractions for PE Part and 4 fractions for PP Phase; (d) Fractionation Program (Single-fraction Protocol) designed for the calculation of the main types of Polyolefins: HDPE and PP, which uses 4 cycles of temperature teatments and results in 2 fractions, One for the PP Part and the second one for HDPE[Fn sch5-fn1]

a
Schemes 5a and 5b are adapted with permission from ref 5. Copyright 2020 Elsevier. Schemes 5c and 5d are adapted from ref 21. Available under a CC-BY 4.0 license. Copyright 2022 John Wiley and Sons.

This approach ingeniously links two independent thermal treatments within a single DSC experiment by exploiting the large difference in melting temperatures between PP (∼165–170 °C) and PE (∼110–130 °C). The methodology unfolds in two consecutive stages, illustrated in Scheme 5a and Scheme 5b.

##### 3.5.1.1 Stage I: Coupled SN Calibration (Scheme 5a)

Before performing the SSA fractionation, Carmeli et al.^5^ first carried out a preliminary coupled SN experiment to determine the *T*_*s,ideal*_ of each component within the blend itself, rather than using the values from the neat polymers. The experiment begins with the SN of the PP phase while the PE remains molten. By progressively heating to different *T*_*s*_^*PP*^ values, and monitoring the shift in *T*_*c*_, the *T*_*s, ideal*_^*PP*^, the highest temperature that still preserves self-nuclei without annealing, is identified at 165 °C. Immediately afterward, the sample undergoes the SN of the PE phase, while the PP remains crystalline. This step yields the *T*_*s,ideal*_^*PE*^ = 127 °C under the realistic interfacial conditions imposed by the solid PP matrix. Thus, Scheme 5a serves as a thermal calibration map, establishing the two reference temperatures that will define the subsequent SSA protocol.

##### 3.5.1.2 Stage II: Coupled SSA Fractionation (Scheme 5b)

Once the *T*_*s, ideal*_ are defined, the coupled SSA fractionation is performed, as shown in Scheme 5b. The program begins with the PP phase, starting at *T*_*s,ideal*_^*PP*^ = 165 °C and descending in Δ*T*_*s*_ = 7.5 °C intervals (165 → 157.5 → 150 → 142.5 → 135 °C), generating four PP fractions (1–4). The use of different fractionation windows for PP (Δ*T*_*s*_ = 7.5 °C) and PE (Δ*T*_*s*_ = 5 °C) is dictated by their distinct SSA fractionation behavior and lamellar stability distributions. In SSA, Δ*T*_*s*_ is selected to generate well-defined, nonoverlapping thermal fractions rather than to simply span a melting interval. In this context, the PP phase, characterized by a limited number of dominant lamellar populations, can be adequately resolved using larger temperature steps, whereas polyethylene requires finer fractionation to separate HDPE- and LDPE-like lamellae without loss of resolution or artificial merging of adjacent fractions.

Instead of executing a final heating for PP, the cycle continues directly with the PE block, initiated at *T*_*s,ideal*_^*PE*^ = 127 °C and descending in Δ*T*_*s*_ = 5 °C steps (127 → 122 → 117 → ··· → 87 °C), producing eight PE fractions (5–12). Both sequences are conducted with a scanning rate of 10 °C/min and *t*_*s*_ = 5 min per step, leading to a total of 14 thermal cycles (5 for PP + 9 for PE).

Finally, a single heating scan melts all crystalline populations, yielding a composite SSA profile that contains the full melting hierarchy of both phases. In these experiments, the highest temperature (225 °C) erased the thermal history of PP, while the lowest (20 °C) defined the standard crystalline state. The coupled arrangement shortened total analysis time by approximately 30% compared to performing separate experiments for each polymer.

The resulting SSA endotherms, exemplified in Figure 20a, show the clear separation of PP-related fractions (1–4) from PE-related fractions (5–12). The first group corresponds to the more stable PP lamellae, while fractions 5–7 are associated with HDPE-like crystallites and fractions 8–12 with LDPE-like chains.

**Figure 20:**
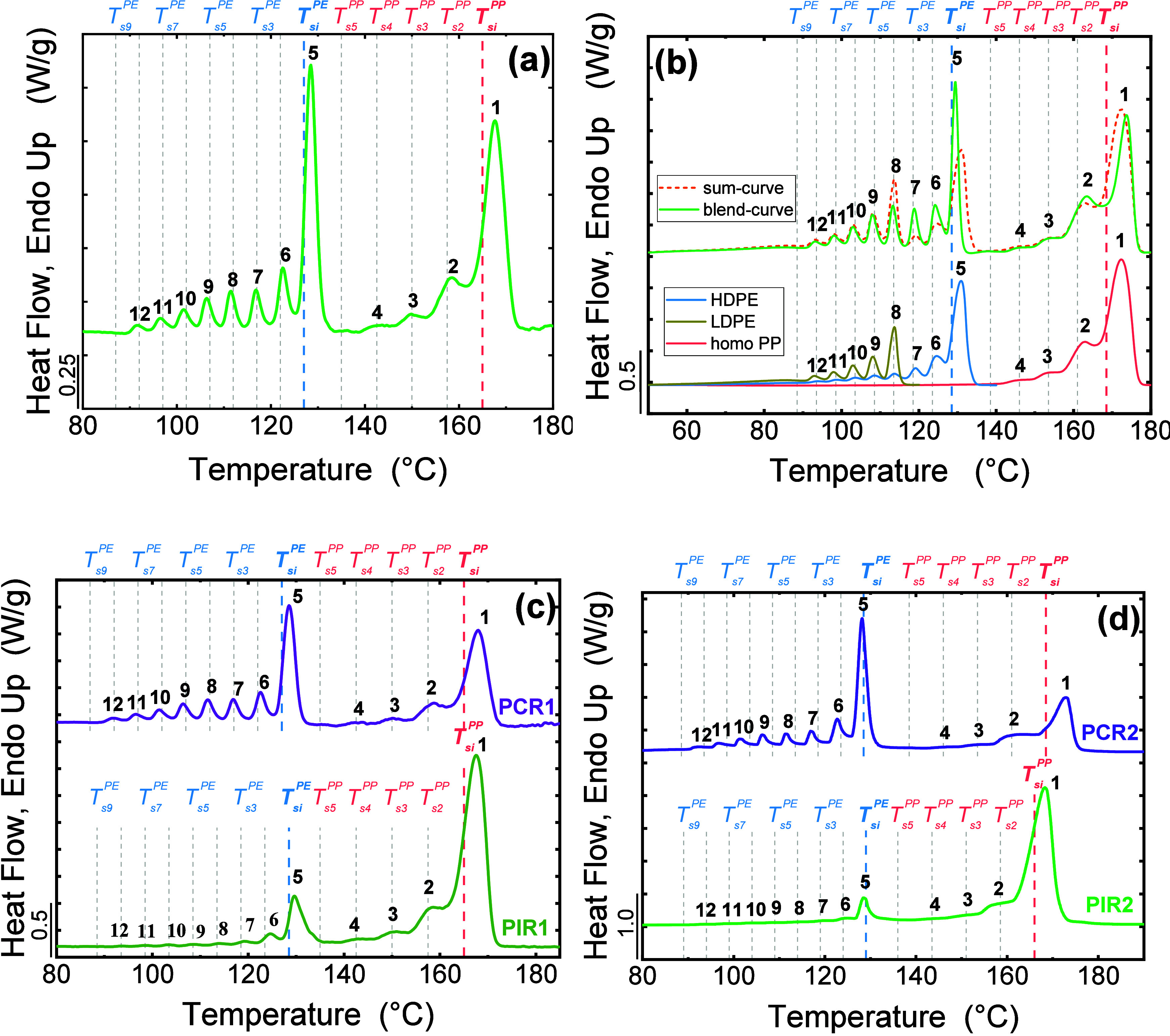
(a) Final heating scan of the couple SSA protocol applied to PCR1, using Δ*T*_*s*_^PE^ = 5 °C and Δ*T*_*s*_^PP^ = 7.5 °C. (b) Comparison between the blend-curve and the sum-curve for the 40/60homo and SSA final DSC heating scans measured for each model component, scaled with their specific concentration in the blend. (c) Comparisons of PCR1 and PIR1 and (d) PCR2 and PIR2. In Figure 20, the dashed vertical lines correspond to the employed values of *T*_*si*_ and *T*_*s*_ for each phase. Numbers 1–4 and 5–12 are assigned to the PP and PE fractions. The *T*_*s*_ lines for individual components (HDPE, LDPE, and homo PP) are not shown. Figure 20 is adapted with permission from ref 5. Copyright 2020 Elsevier.

As illustrated in Figure 20b for the 40/60 homo blend, deviations between the blend-curve (experimental) and the sum-curve (constructed from neat components) provide evidence of cocrystallization and diluent effects. In particular, the lowering of *T*_*m,SSA*_ in peaks 6–8 reflects the cocrystallization of long-chain LDPE sequences with HDPE lamellae, whereas the stronger depression of peak 5 (at *T*_*s*_^*PE*^ ∼ 123.5 °C) reveals the diluent effect of molten LDPE chains on HDPE crystallization. Minor shifts in the PP region (fractions 1–4) are attributed to a slightly lower *T*_*s,ideal*_^*PP*^ in the blends (∼163.5 °C) compared to the neat PP (∼168.5 °C).

Importantly, SSA does not rely solely on melting temperature differences to distinguish polyethylene types. Instead, it exploits differences in lamellar stability and crystallizable sequence length, which translate into distinct high-temperature fraction distributions for HDPE, LLDPE, and LDPE. This approach enables their differentiation in a calibration-based manner through SSA peak areas even when their conventional DSC melting ranges largely overlap.

Quantitative determination of each phase was achieved using two straightforward relationships (eqs 1 and 2) based on the relative areas of the SSA melting peaks:

$$W_{\mathrm{HDPE}}=\frac{A_{5}^{\mathrm{blend}}}{A_{5}^{\mathrm{HDPE}}}$$ 1

$$W_{\mathrm{PP}}=\frac{A_{\mathrm{PPtot}}^{\mathrm{blend}}}{A_{\mathrm{PPtot}}^{\mathrm{PP}}}$$ 2

where *A*_5_^blend^ and *A*_5_^HDPE^ are the areas of the HDPE-like fraction (peak 5) in the blend and the neat reference, respectively, and *A*_PPtot_^blend^ and *A*_PPtot_^PP^ correspond to the sum of PP fractions 1–4. The compositions obtained by these eqs (Table 2) deviated by less than ± 13% from those determined by TREF, confirming SSA as a reliable quantitative tool for mixed polyolefins. The relative uncertainty was estimated at ± 12.7%, as verified by TREF measurements (Table 2).

**Table 2:** Composition of PIR2 and PCR2 Determined by TREF and DSC/SSA^a^

**component**	**PIR2**		**PCR2**	
	**TREF**	**SSA**	**TREF**	**SSA**
PP	82.4	84	41.4	41
HDPE	7.6	7	24.5	30
LDPE(+VLDPE)			26.3	
soluble fraction	10		7.8	

a The values are weight percentages. Table based on ref 5.

Applying these eqs (eqs 1 and 2), Carmeli et al.^5^ calculated the composition of the real recycled blends (PCR and PIR samples), whose SSA profiles are shown in Figure 20c and Figure 20d. For PCR1 and PIR1 (nominally 40/60 and 10/90 PE/PP), shown in Figure 20c, the SSA results yielded *W*_*HDPE*_ = 21% and 11%, respectively, consistent with the expected higher proportion of HDPE-like material in PCR1. Considering the total PE phase, this implies that the PE in PIR1 is almost exclusively HDPE, whereas in PCR1, approximately half is HDPE and the remainder LDPE.

For the unknown composition samples, PCR2 and PIR2, shown in Figure 20d, the estimated compositions were 41% PP + 30% HDPE-like +29% others and 84% PP + 7% HDPE-like +9% others, respectively.

The remaining components likely include LDPE-like chains, fillers, or undetectable solubles. Comparison with TREF confirmed excellent agreement between both methods (Table 2).

#### 3.5.2 Protocol Simplification: The Single-Fraction Method

While the coupled SN + SSA procedure provided detailed compositional maps, its 14 thermal cycles were too time-consuming for routine or industrial analyses.

To overcome this limitation, Góra et al.^21^ revisited the original data set of Carmeli et al.^5^ and noted that most quantitative information was concentrated in two dominant melting fractions, the first PP fraction (Fraction 1, ∼160–170 °C) and the first HDPE-like fraction (Fraction 5, ∼125–132 °C). By correlating the enthalpy of these two peaks with the total PP and HDPE contents obtained from the complete 14-cycle SSA and from TREF, they observed an almost perfect linear relationship (R^2^ = 0.98). This demonstrated that the two peaks could serve as reliable quantitative fingerprints for the respective polymer phases.

To verify this hypothesis, Góra et al.^21^ applied a simplified “single-fraction” program to a broad series of model PE/PP blends and commercial recyclates. The model blends combined PP homopolymer with HDPE (ρ = 0.945 g/cm^3^) and LDPE (ρ = 0.923 g/cm^3^) in five compositions spanning the full range, 80/20, 65/35, 50/50, 35/65, and 20/80 (PE/PP), while maintaining a 50/50 ratio of HDPE and LDPE within the PE phase.

The simplified protocol, depicted in Scheme 5c and Scheme 5d, consists of only four temperature cycles, two for PP and two for PE, executed consecutively in a single DSC run.

Each polymer undergoes one self-nucleation and one annealing step at its *T*_*s,ideal*_ (*T*_*s,ideal*_^*PP*^ = 165 °C; *T*_*s,ideal*_^*PE*^ = 127 °C), thereby generating a single representative fraction per component. Figure 21a compares the resulting SSA profiles in the 65/35 PE/PP blend, in which both protocols were applied. The faster protocol (Scheme 5d) reproduces the key melting events from the multifraction program, with identical peak positions and relative areas.

**Figure 21:**
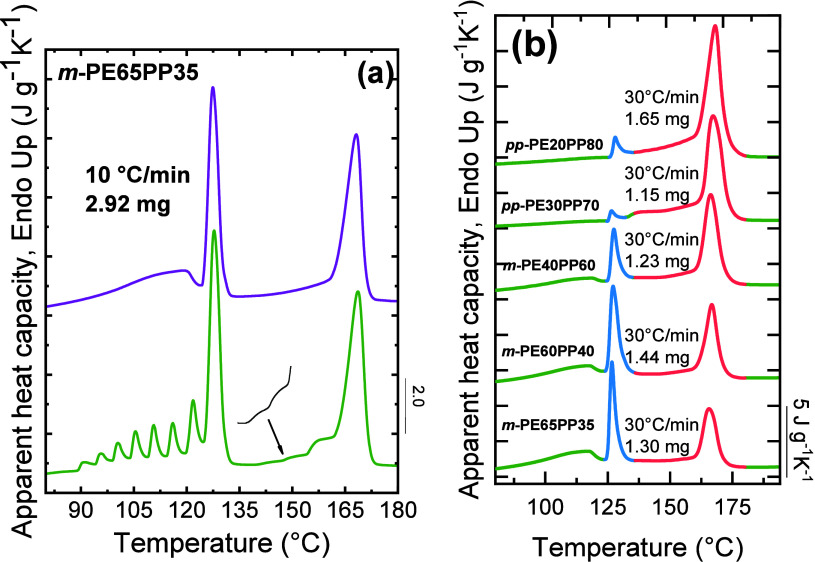
(a) Comparison of the outcome of the two SSA fractionation protocols for m-PE65PP35: the DSC curve in red corresponds to the single-fraction and the one in black to the multifraction protocol. (b) Fractionation output run results for the investigated materials: the content of PP decreases from top to bottom, while that of PE correspondingly increases in the same direction. Figure 21 is adapted from ref 21. Available under a CC-BY 4.0 license. Copyright 2022 John Wiley and Sons.

To validate the approach across composition, Góra et al.^21^ analyzed a complete series of model PE/PP blends, 80/20, 65/35, 50/50, 35/65, and 20/80 (PE/PP). The corresponding single-fraction scans, displayed in Figure 21b, reveal the systematic evolution of the two characteristic peaks: the PP peak (∼165 °C) increases in intensity as PP content rises, whereas the PE peak (∼125 °C) becomes dominant in PE-rich systems. The relative areas of these two peaks scale linearly with composition, confirming that they can serve as quantitative fingerprints for both polymer phases.

Quantitative evaluation using eqs 1 and 2 yielded composition values within ± 3% of those determined by TREF for all blends and recycled samples, confirming that the reduced experiment retains full analytical reliability.

In addition, Góra et al.^21^ demonstrated that the scan rate could be increased from 10 °C/min to 30 °C/min, provided the sample mass was proportionally decreased (2.9 → 1.3 mg) to maintain equivalent thermal equilibration. This modification reduced total analysis time from ∼420 min for the complete 14-cycle protocol to ∼75 min, without compromising precision.

The single-fraction SSA protocol therefore established a fast, reproducible, and industry-friendly route for the quantitative screening of recycled polyolefin blends across a wide compositional spectrum.

#### 3.5.3 Quantitative Analysis of Commercial Post-consumer Recycled Blends

Building upon the coupled and accelerated SSA protocols described above, Coba-Daza et al.^42^ applied the method to postconsumer recycled PE blends containing LDPE, LLDPE, and minor PP contamination. The aim was to evaluate whether SSA could deliver reliable compositional data in heterogeneous recyclates, where conventional DSC analysis often fails due to overlapping melting events and cocrystallization between polyethylene components.

The study combined a set of neat reference materials, model blends, and commercial recyclates. Neat LDPE and four LLDPE grades of increasing density (ρ = 918, 923, 931, 935 kg/m^3^) were used to span different short-chain branching levels. Model LDPE/LLDPE blends (70/30, 60/40, 50/50 wt %) were prepared from virgin materials, and six postconsumer resins (REC_0_-REC_5_) served as unknowns.

A unified SSA protocol was implemented to ensure quantitative comparability among all samples. Following Müller et al.’s^3,4^ recommendations, the highest *T*_*s*_ was fixed at *T*_*s*_ = 128 °C (*Domain II* for the set), combined with a constant fractionation window of Δ*T*_*s*_ = 5 °C and an isothermal time of *t*_*s*_ = 5 min per step. Twelve successive steps were applied (128 → 73 °C), producing coincident valley positions across the SSA heating traces. This identical thermal history allowed direct comparison of peak areas between neat, model, and recycled samples, while preventing any annealing events.

Figure 22 displays representative SSA final-heating scans for neat LDPE and LLDPE (Figure 22a), “unmixed” (Figure 22b) theoretical blends (constructed as weighted sums of the neat traces), model experimental blends (Figure 22c), and a representative recycled (Figure 22d) material (REC_1_).

**Figure 22:**
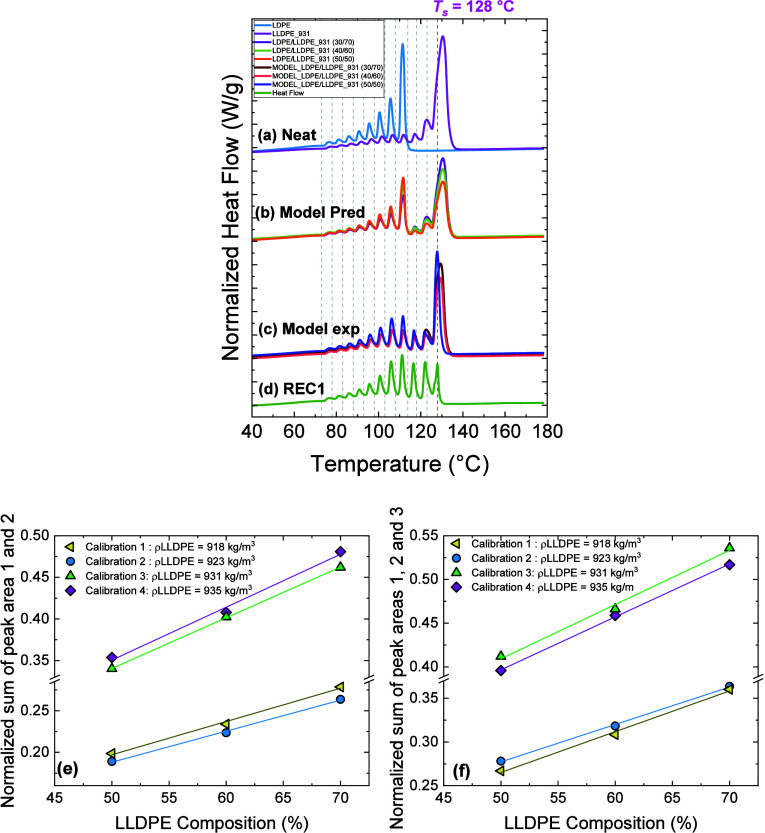
SSA profiles for (a) neat LDPE and LLDPE, (b) sum of neat curves at different compositions to create the model predicted, (c) experimental model blends at different compositions, and (d) resulting DSC scan from recycled material number 1. (e) Calibration curves obtained from the model DSC experimental curves considering the first two peaks and (f) considering the first three peaks from the SSA fractionation melting curve. Figure 22 is adapted from ref 42. Available under a CC-BY 4.0 license. Copyright 2024 Springer Nature.

Compared to the unmixed predictions, the model blends show narrower and slightly shifted high-temperature peaks, indicating cocrystallization and diluent effects between LDPE and LLDPE sequences. Recycled materials exhibit broader, asymmetric distributions, reflecting the coexistence of linear and highly branched chain populations typical of mixed feedstocks.

Quantitative determination of the LLDPE fraction was achieved through density-specific calibration curves built from the model blends. For each LLDPE grade, the area of the two or three highest-temperature fractions in the SSA final heating (140 → 119.7 °C and 140 → 114.9 °C, respectively) was integrated and normalized by the total melting area (25 → 140 °C). Plotting these normalized sums against the known LLDPE contents yielded linear correlations (R^2^ > 0.99), with slopes increasing with LLDPE density (Figure 22e,f).

As expected, more linear (higher-density) LLDPEs allocate a larger fraction of their melting enthalpy to the high-temperature region. The two-peak calibration proved most robust since it minimized deviations caused by residual cocrystallization.

For unknown recyclates, Coba-Daza et al.^42^ proposed a simple criterion to identify the most suitable calibration curve: if the area of the highest-temperature fraction (*A*_1_) exceeds 1.5 × *A*_2_ (the next fraction), the recyclate behaves as a higher-density LLDPE and should be compared to the ρ = 931–935 kg/m^3^ calibration; otherwise, the ρ = 918–923 kg/m^3^ set is applied.

This empirical *A*_1_ */A*_2_ rule effectively connects the lamellar stability distribution with the average branching density. When applied to the six postconsumer materials (REC_0_–REC_5_), the SSA-derived LLDPE compositions matched ^13^C NMR values within ± 2% (Figure 23). In contrast, results obtained by temperature-modulated DSC (TMDSC), originally developed for virgin materials, deviated significantly for these complex recyclates.

**Figure 23:**
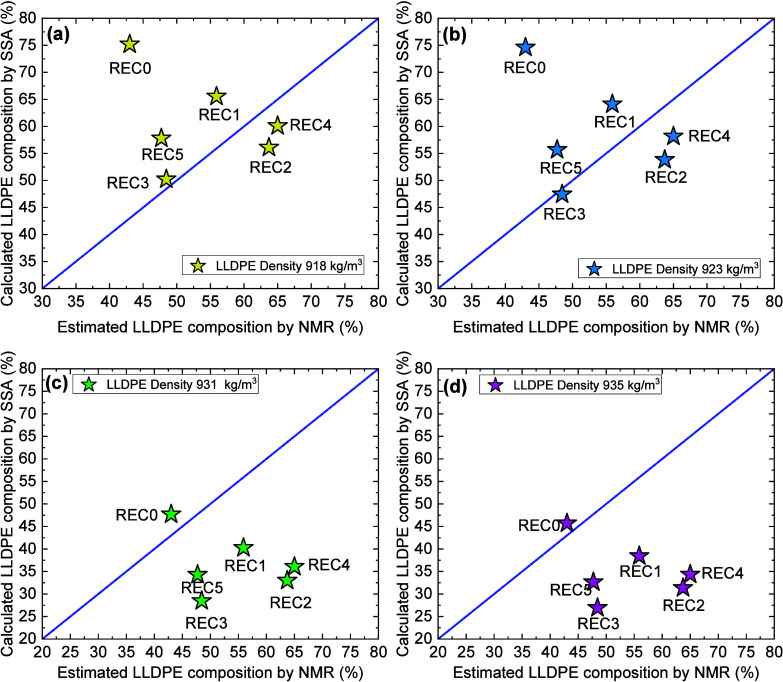
Comparison of the estimated LLDPE composition by NMR and the calculated LLDPE composition by SSA of six different recycled materials, considering the first two areas of the fractionated melting SSA result at different LLDPE densities as (a) 918 kg/m^3^; (b) 923 kg/m^3^ ; (c) 931 kg/m^3^; and (d) 935 kg/m^3^. Figure 23 is adapted from ref 42. Available under a CC-BY 4.0 license. Copyright 2024 Springer Nature.

The high degree of agreement with NMR validates SSA as a precise and reproducible quantitative tool for recycled polyethylene systems.

Although the main SSA workflow focuses on PE composition, trace PP contamination (∼160 °C) can be quantified by a short SN test prior to the SSA run. Annealing the recyclate at *T*_*s*_ ∼ 160 °C (*Domain III* for PP) yields a distinct PP endotherm separated from the PE melting zone, allowing its estimation via area-ratio principles, similar to those in eqs 1 and 2.

Combining this SN step with the SSA fractionation, therefore, provides a comprehensive thermal fingerprint of postconsumer polyolefin blends. The unified SSA protocol (*T*_*s*_ = 128 °C; Δ*T*_*s*_ = 5 °C; 12 steps) enables reproducible quantification of LDPE/LLDPE composition and chain linearity in complex recycled materials. In this case, by correlating normalized high-temperature fraction areas with calibration curves derived from model blends, SSA delivers NMR-level compositional accuracy using only standard DSC equipment. This study represents a practical step toward the potential use of SSA as a fast, solvent-free quality-control tool for real industrial recycling streams.

As with any crystallization-based technique, the presence of strong nucleating agents, fillers, pigments, or uncontrolled impurities in recycled materials may modify nucleation density and lamellar development, potentially affecting SSA fractionation profiles. For this reason, quantitative SSA analysis is most reliable when calibration curves are constructed from representative reference materials processed under comparable conditions, and when SSA results are interpreted alongside complementary compositional or structural techniques. Rather than a limitation of SSA alone, this requirement reflects the intrinsic complexity of real recycled feedstocks.

#### 3.5.4 Monitoring Lamellar Reorganization during Mechanical Recycling

Mechanical recycling often induces competing molecular processes, chain scission, re-entanglement, and recrystallization, that alter the crystalline morphology of semicrystalline polymers. While conventional DSC can detect overall changes in crystallinity, it lacks the resolution to track the redistribution of lamellar populations associated with these structural transformations. In this context, Morales et al.^45^ demonstrated that SSA provides a powerful means to monitor the evolution of lamellar thickness and crystal perfection in a biobased polyamide 11 (PA11) and its PA11/LDPE (90/10) recycled blends over multiple mechanical reprocessing cycles.

Virgin PA11 and its LDPE blend were subjected to ten extrusion–grinding recycling cycles under identical processing conditions. Samples were taken after each cycle, and standard DSC and SSA were used to analyze their crystallization and melting behavior. The SSA experiments were carried out using a *T*_*s,ideal*_ = 176 °C, selected from SN tests that defined Domain II for the neat PA11, and a Δ*T*_*s*_ = 5 °C with a *t*_*s*_ = 5 min, resulting in ten successive fractionation steps (176 → 131 °C). The same parameters were applied to the PA11/LDPE blend to ensure comparability. This uniform protocol allowed the evolution of the lamellar distributions to be followed directly as a function of recycling history.

Figure 24 shows representative SSA final-heating curves for virgin and recycled samples.

**Figure 24:**
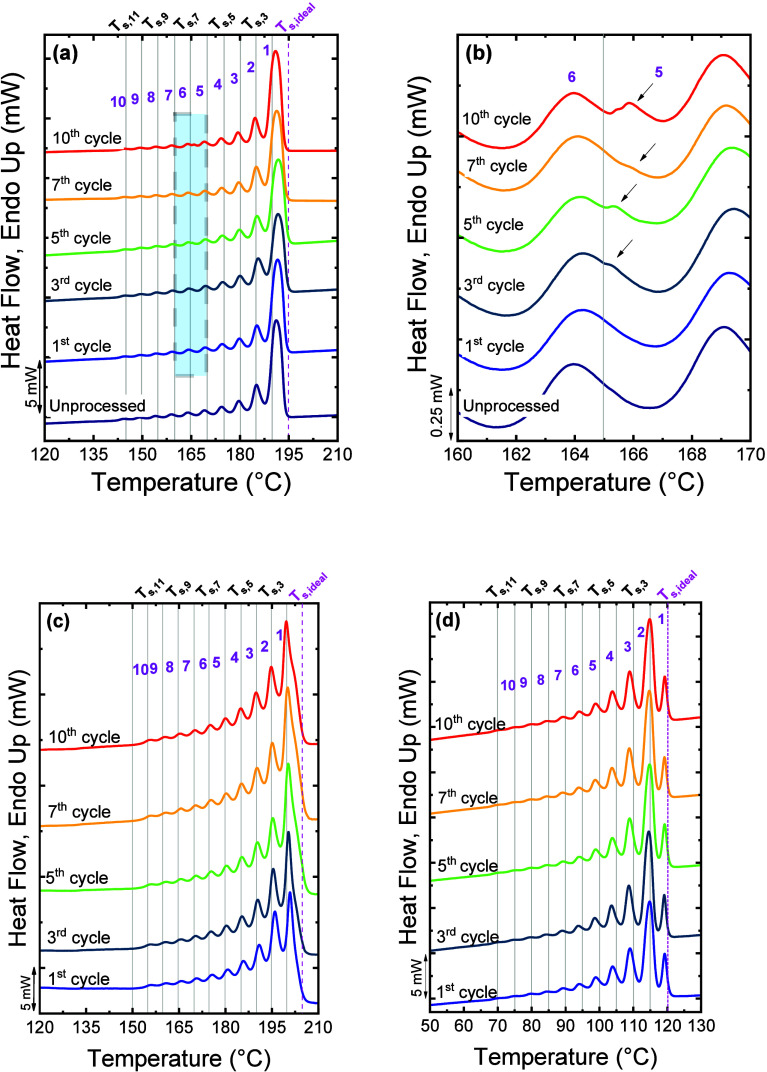
SSA profiles for (a) virgin PA11 and (b) magnifications of peaks 5 and 6 of virgin PA11 (from Figure 24a, see shadowed region), (c) postconsumer PA11, and (d) LDPE in the postconsumer PA11. Figure 24 is adapted from ref 45. Available under a CC-BY 4.0 license. Copyright 2025 John Wiley and Sons.

The virgin PA11 exhibits a single dominant melting population at ∼190 °C (Figure 24a), corresponding to well-defined α-form lamellae. After several recycling cycles, an additional low-temperature shoulder (∼170–175 °C) appears (Figure 24a,b) and gradually increases in intensity (Figure 25a), while the main peak slightly narrows and shifts to lower temperatures. This behavior indicates the progressive formation of thinner, less perfect lamellae due to partial chain scission and redistribution of the crystalline fraction during repeated processing. Although total crystallinity remains almost unchanged, the lamellar hierarchy evolves, indicating that SSA is more sensitive to morphological redistribution than to the amount of crystalline phase alone.

**Figure 25:**
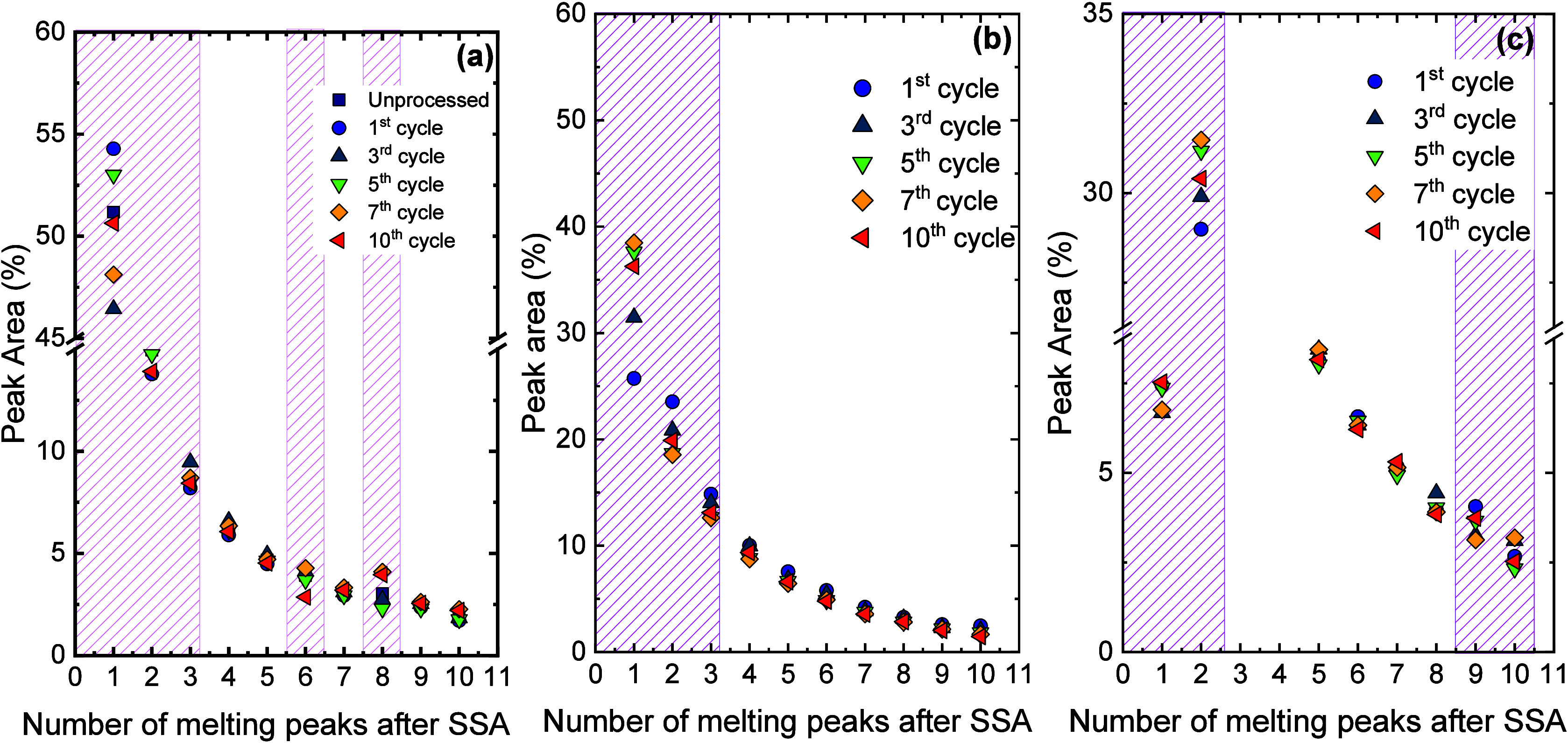
Peak area after SSA for (a) virgin PA11 and (b) postconsumer PA11 and (c) LDPE in the postconsumer PA11. Figure 25 is adapted from ref 45. Available under a CC-BY 4.0 license. Copyright 2025 John Wiley and Sons.

The PA11/LDPE blend (Figure 24c,d) exhibits two distinct melting domains in the SSA final heating: (i) a broad low-temperature region (∼110–120 °C) associated with LDPE (Figure 24d), and (ii) the main α-form PA11 peak (∼190 °C), see Figure 24c.

During the first recycling cycles, both phases can be individually fractionated within the same SSA program: the LDPE domain produces a series of low-*T*_*m*_ fractions separated by 5 °C, while the PA11 phase displays its characteristic sequence of higher-temperature fractions. This dual-phase fractionation confirms that SSA can deconvolute independent crystalline populations even in partially immiscible systems.

As the number of recycling cycles increases, the LDPE fraction remains almost unchanged in position and intensity, indicating that its crystalline network is largely unaffected by processing. Conversely, the PA11 endotherm becomes sharper and slightly shifts to higher temperatures, signifying lamellar thickening and enhanced regularity. This evolution, better visualized in Figure 25b and Figure 25c, which plots peak areas vs the number of fractions, suggests that the dispersed LDPE phase acts as a stabilizing and heterogeneous nucleating agent, mitigating PA11 degradation and facilitating lamellar reorganization at the interface. Thus, the blend preserves its crystalline integrity after multiple reprocessing cycles, whereas the neat polymer forms thinner lamellae.

The contrasting SSA responses of neat PA11 and the PA11/LDPE blend highlight SSA’s diagnostic capability for evaluating structural stability in recycled biopolymers. In neat PA11, the growth of a low-*T*_*m*_ fraction reflects molecular degradation and lamellar subdivision, whereas in the blend, the stabilization of both PA11 and LDPE crystalline domains demonstrates that interfacial nucleation can counteract morphological degradation. SSA thereby provides a quantitative, mechanistic picture of how processing history affects lamellar organization, information inaccessible to conventional DSC.

Importantly, this case also highlights that extending SSA to mechanically recycled polar engineering polymers is nontrivial. Polyamides combine strong intermolecular interactions (e.g., hydrogen bonding), complex lamellar reorganization pathways, and a high sensitivity to processing-induced changes in molecular weight, end-group chemistry, and nucleation density. In this context, SSA should be regarded primarily as a tool to track the redistribution and stability of lamellar populations rather than as a direct quantitative probe of a single structural parameter. In recycled PA11 systems, multiple effects, including chain scission, re-entanglement, changes in heterogeneous nucleation, and interfacial stabilization by a dispersed polyolefin phase, can contribute simultaneously to the observed SSA profiles. Precisely because of this complexity, SSA offers a unique advantage: it resolves how these competing mechanisms reshape the lamellar hierarchy, even when overall crystallinity remains nearly unchanged in conventional DSC.

Overall, Morales et al.^45^ established SSA as a sensitive tool for tracking lamellar reorganization during mechanical recycling, extending its utility beyond compositional analysis to the assessment of morphological durability in biobased semicrystalline polymers.

## 4 Concluding Remarks and Future Perspective

The past decade has seen a remarkable expansion of SSA applications across the polymer landscape. As summarized in Table 1, which compiles ∼130 studies (2015–2025) organized into three broad categories: (i) sustainable, biodegradable, and recycled materials; (ii) polyolefins; and (iii) other emerging semicrystalline systems, the technique has become an essential tool for probing crystallization phenomena in modern materials.

Across these systems, SSA consistently reveals structural information that standard nonisothermal DSC experiments cannot capture, even when performed on the same instrument. In homopolymers, SSA reveals the influence of intermolecular interactions, branching, and topology on lamellar distributions and attainable melting temperatures that are closer to thermodynamic equilibrium values. In random copolymers, SSA provides crystallization fingerprints: unimodal, bimodal, or shifted melting sequences that reflect comonomer inclusion/exclusion balances, pseudoeutectic compositions, and hidden mixed modes. In nanocomposites, SSA resolves hierarchies of interfacial crystallization, distinguishing between fractionable supernucleated lamellae, oriented interfacial crystals, prefrozen layers above *T*_*m*_°, and adsorbed or intercalated domains. In more complex architectures (triblock terpolymers, TPUs, multifunctional systems), SSA clarifies overlapping transitions, enhances the detection of subtle solid–solid transformations, and identifies block-specific crystalline populations. Finally, in recycled materials, SSA quantifies PP/PE compositions and branching distributions and tracks lamellar reorganization during mechanical reprocessing, offering mechanistic insight into degradation and re-entanglement phenomena.

At the same time, the broad applicability of SSA also entails intrinsic limitations that must be carefully considered. In many practical systems, particularly complex copolymers, multiphase materials, nanocomposites, and recycled polymers, SSA melting or crystallization peaks may partially or strongly overlap, complicating their direct physical interpretation. In such cases, the overlap reflects the coexistence of multiple crystalline populations with comparable thermal stability, which hinders the unambiguous assignment of individual lamellar fractions. As a result, SSA profiles in these systems should be interpreted primarily in a comparative manner, focusing on relative changes under identical and well-defined thermal protocols rather than as unique or absolute descriptors of lamellar distributions.

More generally, SSA is most robust when used for qualitative comparison and relative ranking of samples analyzed under controlled and reproducible conditions. Nevertheless, it should be emphasized that a genuinely quantitative level of SSA analysis has been achieved in well-established material families, most notably polyolefins, where decades of accumulated knowledge have firmly established the relationships between SSA fractionation profiles, crystallizable sequence length, lamellar thickness, and short-chain branching distributions. In these mature systems, quantitative SSA analysis can now be performed using standardized protocols without the need for systematic cross-validation in every individual study. In contrast, for emerging, chemically complex, or less-studied materials, this level of methodological maturity has not yet been reached; in such cases, SSA should be applied primarily as a comparative or semiquantitative tool, and quantitative interpretations should be supported by complementary experimental evidence.

Looking ahead, SSA is poised for even broader adoption. A significant milestone is the new ISO standard (2025) for determining short-chain branching distributions in ethylene/α-olefin copolymers using SSA, marking its transition from an academic protocol to an industry-recognized quality-control method. Technically, coupling SSA with ultrafast calorimetry opens the door to high-throughput screening and the study of early stage fractionation kinetics. Integration with operando structural techniques (WAXS/SAXS, FT-IR, AFM, rheology) will provide real-time insight into polymorphic transitions, interfacial ordering, and metastable crystal populations. As the large data set summarized in Table 1 continues to grow, data-driven and machine-learning approaches are expected to transform SSA melting profiles into predictive fingerprints of composition, crystallization mode, and processing history.

Taken together, SSA now stands as more than a thermal fractionation method: it is an emerging conceptual and practical framework connecting molecular structure, crystallization pathways, recyclability, and sustainable polymer design.
